# Collagen Type I as a Biological Barrier Interface in Biomimetic Microfluidic Devices: Properties, Applications, and Challenges

**DOI:** 10.3390/biomimetics11010066

**Published:** 2026-01-13

**Authors:** Valentina Grumezescu, Liviu Duta

**Affiliations:** National Institute for Laser, Plasma and Radiation Physics, 077125 Magurele, Romania

**Keywords:** collagen type I, biological barrier interface, biomimetic microfluidic devices, TEER, “on-chip” technological readiness

## Abstract

Collagen type I has become a practical cornerstone for constructing biologically meaningful barrier interfaces in microfluidic systems. Its fibrillar architecture, native ligand display, and susceptibility to cell-mediated remodeling support epithelial and endothelial polarization, tight junctions, and transport behaviors that are difficult to achieve with purely synthetic barrier interfaces. Recent advances pair these biological strengths with tighter engineering control. For example, ultrathin collagen barriers (tens of micrometers or less) enable faster molecular exchange and short-range signaling; gentle crosslinking and composite designs limit gel compaction and delamination under flow; and patterning/bioprinting introduce alignment, graded porosity, and robust integration into device geometries. Applications now span intestine, vasculature, skin, airway, kidney, and tumor–stroma interfaces, with readouts including transepithelial/transendothelial electrical resistance (TEER), tracer permeability, and image-based quality control of fiber architecture. Persistent constraints include batch variability, long-term mechanical drift, limited standardization of fibrillogenesis conditions, and difficulties scaling fabrication without loss of bioactivity. Priorities include reporting standards for microstructure and residual crosslinker, chips for continuous monitoring, immune-competent co-cultures, and closer collaboration across materials science, microfabrication, computational modelling, and clinical pharmacology. Thus, this review synthesizes the state-of-the-art and offers practical guidance on technological readiness and future directions for using collagen type I as a biological barrier interface in biomimetic microfluidic systems.

## 1. Introduction

Microfluidic devices have rapidly evolved as transformative platforms in biomedical research, owing to their ability to recreate complex physiological microenvironments in controlled in vitro conditions. These systems, which involve the manipulation of fluids at the microscale, are increasingly used in tissue engineering, drug testing, and organ-on-chip technologies. Thus, in tissue engineering, microfluidic platforms enable controlled cell culture conditions and dynamic nutrient exchange, which are crucial for maintaining viable and functional three-dimensional constructs [1]. In drug discovery and testing, these devices allow the assessment of toxicity in microphysiological systems (MPSs), often surpassing traditional static culture assays in predictive power [2,3]. Organ-on-chip systems represent a significant breakthrough in translating preclinical in vitro studies toward clinical relevance, allowing the recapitulation of human organ physiology on a micro-engineered device. Thereby, mechanistic studies and drug screening with improved predictability compared to conventional approaches are facilitated [4,5,6].

Central to these systems is the barrier interface that separates compartments, mimicking tissue-tissue interfaces such as the intestinal wall, blood–brain barrier (BBB), or tumor microenvironment. Barrier interfaces are pivotal components of organ-on-a-chip (OoC) platforms, as they provide a structural substrate for adherent cells, regulate the bidirectional exchange of nutrients, metabolites, and signaling molecules through defined pores, and serve as interfaces for the controlled delivery of mechanical and chemical stimuli [7].

Traditionally, microfluidic barrier models have been built on synthetic interfaces such as polyethylene terephthalate (PET), polycarbonate (PC), and polydimethylsiloxane (PDMS). These materials are appealing for their mechanical stability, low chemical reactivity, favorable thermal properties, ease of fabrication, robustness and flexibility, optical transparency, and general biocompatibility [7,8,9]. In recent years, thermoplastics have gained traction as alternatives to PDMS and other legacy substrates, offering improved functionality, reliability, and scalability [10]. Common choices for microfluidic systems include polyurethane methacrylate (PUMA), elastomers such as thermosetting polyester (TPE), poly(methyl methacrylate) (PMMA), cyclic olefin polymer (COP), PC, and polystyrene (PS) [11]. Many of these platforms still face practical constrains—higher fabrication costs, limited long-term durability, lengthy processing, and multistep, equipment-intensive workflows—that complicate manufacturing at scale [12]. More important, synthetic barrier interfaces rarely capture the biochemical and biomechanical richness of native extracellular matrix (ECM), which limits itsability to recapitulate tissue-specific cues and cell–matrix interactions.

In recent years, increasing attention has been directed toward the development of biological barrier interfaces that can better mimic physiological conditions. Unlike their synthetic counterparts, biological barrier interfaces possess intrinsic biochemical signaling factors and mechanical properties that promote appropriate cell adhesion, polarization, and differentiation [6,13]. These features are critical for constructing biomimetic models of tissues such as the intestinal wall, alveolar-capillary interface, or BBB. By providing a more faithful recreation of the native microenvironment, biological barrier interfaces enhance the predictive capacity of microfluidic models for both fundamental biology and applied biomedical research [4,13].

The shift from synthetic to natural polymers in barrier interface design is motivated by the need for materials that combine biocompatibility, biodegradability, and biofunctionality. Naturally derived polymers such as gelatin, chitosan, fibrin, and collagen provide cell adhesion sites and are recognized by cellular receptors, which enhances the physiological relevance of tissue engineered constructs [14]. Among these, collagen type I stands out as the most widely used polymer due to its abundance in nature (being the primary constituent of the mammalian ECM), well-established biodegradability, and versatility [15]. As a natural ECM protein, its ability to form fibrillar structures, its tunable mechanical characteristics, intrinsic bioactivity, biocompatibility, biodegradability, and permeability make it a promising candidate for constructing barrier interfaces in microfluidic devices [16].

The prominence of collagen type I in biomedical applications also derives from its integrin-binding domains, which promote cell adhesion and facilitate processes such as migration, differentiation, and polarization [17]. Its enzymatic degradability allows dynamic remodeling, enabling the study of cell–matrix interactions under both physiological and pathological conditions. Furthermore, the capacity of collagen to bind growth factors (such as bone morphogenic protein 2, rhBMP-2 [18]) and modulate biochemical gradients adds another layer of functionality, making it indispensable in the design of biomimetic barrier systems [19]. Last, but not least, it is also important to emphasize that recent advancements in collagen-based microfluidic platforms demonstrate growing interest in using this biomaterial to develop more physiologically relevant in vitro models [20].

From a biomimetic perspective, biological barrier interfaces are pivotal for achieving fidelity in OoC systems. These platforms (‘on-a-chip’) advance physiologically relevant, organ-like architecture by embedding human cells within three-dimensional (3D) microfluidic devices, and they can be configured to isolate discrete functional units—useful when the goal is to probe a specific tissue compartment [21]. More broadly, biomimetics aims to reproduce key structures and functions of living systems in engineered settings, narrowing the gap between in vitro assays and in vivo models. Collagen-based barrier interfaces align perfectly with this approach. Owing to their close structural similarity with native ECM, they enable interfaces that do more than physically separate microfluidic chambers: they also deliver the biochemical and biomechanical cues required to support authentic cell–matrix interactions [22,23].

The aim of this review is to comprehensively analyze the recent strategies for utilizing collagen type I as a biological barrier interface in biomimetic microfluidic systems. We focus on its structural properties, integration methods, functional roles, applications, and the challenges that must be addressed for future translational and clinical applications.

## 2. Literature Review

Given the extensive scope and multidisciplinary nature of collagen research, especially as a barrier interface in microfluidic devices, which spans fields such as biomaterials, tissue engineering, regenerative medicine, and pharmaceutical applications, a comprehensive review of the entire body of literature is neither feasible nor practical within a single manuscript. To ensure focus, relevance, and currency, the present review intentionally concentrates on literature published within the 2023–2025 timeframe. This decision demonstrates a targeted effort to capture the most recent advances, innovations, and emerging trends in collagen research and its biomedical applications.

Rather than applying rigid keyword-based searches—which often risk either omitting significant interdisciplinary studies or returning an unmanageable volume of data—a conceptual and thematic segmentation approach was adopted. In this context, several specific sections were proposed based on emerging research directions, technological developments, and application-driven demands. Within each section, relevant recent publications were critically selected through a combination of expert-driven exploration, manual screening of high-impact journals offering either open access or Institutional full-text availability, and contextual alignment with the predefined thematic areas. This strategy allows for a more curated and insightful synthesis of cutting-edge findings, while still maintaining scientific rigor and transparency.

## 3. Collagen Type I

### 3.1. Molecular Structure and Physical Properties of Collagen Type I

Collagen (type I) is a structural protein present throughout the human body, composed of two α1(I) chains and one α2(I) chain that form a right-handed triple helix, assembling into fibrils and fibers with characteristic D-banding periodicity (~67 nm) [24,25]. Its mechanical and transport properties are influenced by hydration and crosslinking [26,27]. This hierarchical molecular architecture—with defined gap and overlap regions in the D-period—gives rise not just to tensile strength but also to directional transport pathways, since the packing density and molecular staggering influence porosity at the nanoscale. It is important to mention that, in microfluidic or barrier interfaces, preserving the triple-helical conformation and D-band periodicity is critical for reproducible permeability and mechanical stability under flow or wet conditions.

Recent studies deepen insight into the modulation of physical behavior relevant to barrier interface function by means of hydration state. For example, Bhattacharya & Dubey [28] carried out molecular dynamics simulations of collagen type I interfaced with hyaluronan at different water concentrations (65% to 75%) in an annulus fibrosus model. They found that increase in water concentration led to interchain sliding, softening of tensile modulus (from ~2.1 GPa down to ~0.66 GPa) and shift in compressive behavior, features that are directly relevant to collagen barrier interfaces when implemented in wet microfluidic environments: higher hydration can increase permeability, but reduces mechanical resistance. Similarly, water has been demonstrated to act as a critical mediator in the self-assembly of collagen monomers. Thus, Giulia Giubertoni et al. [26] examined how water-collagen interactions are altered in isotopic media (i.e., H_2_O vs. D_2_O) and how these affect self-assembly and modulate intermolecular interactions to optimize fibrillogenesis and ultimately influencing the structural and functional properties of the resulting collagen network.

Crosslinking emerges in the recent literature as a key aspect in tuning both swelling (i.e., water uptake) and permeability, which are critical parameters for the use of barrier interfaces in microfluidic devices. In this respect, Ruliffson et al. [29] characterized commercially available methacrylated collagen type I (“PhotoCol^®^”) photo-crosslinked with different photoinitiators (Lithium phenyl-2,4,6-trimethylbenzoylphosphinate, Irgacure 2959, and Ruthenium/Sodium Persulfate). It was demonstrated that the transport of molecules with a molecular weight of approximately 40 kDa was influenced by the degree of crosslinking, while the diffusion of larger molecules was hindered irrespective of crosslinking. In contrast, permeability studies using 10 kDa dextran revealed that permeability coefficients did not differ significantly between uncrosslinked barrier interfaces and those exposed to 90 s of photo-crosslinking. This indicated that the transport of smaller molecules was largely unaffected by crosslinking. Therefore, beyond ensuring adequate substrate permeability, it is essential to consider how mass transport is modulated within fibrotic microenvironments both in vitro and in vivo. Also, Ren et al. [27] compared various chemical crosslinkers (glutaraldehyde - GTA, proanthocyanidins, hexamethylendiisocyanate, and 1-Ethyl-3-(3-dimethylaminopropyl) carbodiimide/N-hydroxysuccinimide) in collagen barrier interfaces and found that chemical cross-linking significantly enhanced the tensile strength and enzymatic resistance (e.g., to collagenase degradation) of collagen barrier interfaces, thereby extending their stability and functional lifespan in vivo. These trade-offs are central when designing barrier interfaces in microfluidic chips, since one must balance permeability (for, e.g., nutrient or analyte diffusion) with mechanical integrity and stability.

### 3.2. Mechanical, Permeability, and Biochemical Properties of Collagen Type I Barrier Interfaces

The mechanical properties of collagen barrier interfaces can be tailored by adjusting composition, crosslinking degree, and processing method. For instance, UV-cured network architecture of atelocollagen barrier interfaces exhibited about two-fold increase in compression modulus when subjected to sequential functionalization with both 4-vinylbenzyl chloride and methacrylic anhydride [30]. This crosslinking strategy also significantly reduced (by three-fold) the swelling ratio, contributing to dimensional stability under hydrated conditions and permeability [30], which represent essential requirements in microfluidic systems [29]. The incorporation of bioglass nanoparticles (BG NPs) significantly improved the mechanical properties of the hydrogels, as demonstrated by an increase in both storage (nine-fold) and loss moduli. Furthermore, the presence of bioactive ions released from the BG NPs induced notable changes in cell proliferation, with increasing BG NPs concentrations correlating with enhanced cellular activity [15]. These findings are critical when translating collagen-based barrier interfaces into microfluidic environments, where precise mechanical integrity and form stability under hydrated conditions are essential for device function and reproducibility.

One should also emphasize that mechanical adaptation is crucial in biomimetic microfluidic devices, where barrier interface resilience to fluid shear and cyclic strain is required. Thus, electrospun nanofiber barrier interfaces integrating collagen with synthetic polymers have demonstrated increased diameter, hydrophilicity, elongation, and surface roughness after crosslinking, alongside reduced pore size volume [19]. These features not only enhance structural integrity but also influence barrier interface compliance, allowing compatibility with physiological strain regimes observed in gut-, lung-, or vessel-on-chip platforms. Moreover, recent strategies employing photo-patternable materials suggest the feasibility of generating stiffness gradients across substrates to replicate soft-hard tissue interfaces, which could be translated to barrier interfaces for spatial mechanical heterogeneity [31].

The importance of matrix stiffness, microarchitecture, and fluid transport dynamics in regulating tumor spheroid morphogenesis was also investigated [32]. Increasing collagen concentration in biomimetic matrices tended to support the formation of larger spheroids in cell lines with more epithelial-like phenotypes. By contrast, although a higher collagen content appeared necessary for mesenchymal cells to self-organize into spheroids, the resulting aggregates were smaller than those formed in lower-collagen matrices. These trends coincided with shifts in the mechanical landscape of the hydrogels—most notably changes in stiffness, microstructural organization, and hydraulic permeability.

The permeability of collagen type I barrier interfaces is predominantly governed by pore size, thickness, hydration state, and crosslinking density [27,30]. ECM-based barrier interfaces often present challenges in reproducibility, particularly when integrated into organ-on-chip platforms requiring consistent diffusion profiles [33,34]. Barrier interfaces in organ-on-chip systems provide cellular environments that closely recapitulate in vivo conditions. These environments are influenced not only by mechanical cues, such as shear stress and cyclic stretching, but also by intrinsic material properties, including stiffness and surface topography. PDMS remains a preferred substrate for stretching applications and gene expression studies, although it typically requires protein coatings to support long-term cell adhesion and functionality. In contrast, ECM-based barrier interfaces inherently contain bioactive proteins, which promote sustained cell growth and more physiologically relevant responses. However, these barrier interfaces often lack mechanical robustness and standardization, limiting their reproducibility and comparative evaluation. Ideally, advanced barrier interfaces should integrate native ECM proteins in architectures that are sufficiently thin to enable direct cell–cell communication and contact, while retaining the flexibility required to deliver controlled mechanical stimulation [7]. Nonetheless, compositional tuning of collagen blends and controlled fabrication, such as freeze-drying or electrospinning, offer routes to modulate molecular sieving behavior across the barrier interface.

The biochemical stability of collagen type I is a key determinant in its long-term functionality as a biological barrier. Enzymatic degradation by collagenase, for instance, has been shown to compromise epithelial barrier integrity in dual-flow microfluidic platforms, evidenced by loss of collagen immunoreactivity, and increased mucosal permeability [35]. This highlights the importance of enzymatic resistance in inflammation-mimicking or tumor-on-chip models. Crosslinking strategies not only modulate mechanical and permeability features but also delay enzymatic degradation, which enhances barrier interfaces durability under proteolytic stress.

Finally, the integration of collagen type I barrier interfaces in microfluidic systems must also consider functionalization with bioactive agents to support cellular adhesion, polarity, and intercellular communication. While such biochemical modulation remains underexplored in standardized OoC barrier interface systems, advances in collagen composite engineering and localized crosslinking open pathways for customized biochemical microenvironments. Collectively, the mechanical, permeability, and biochemical tunability of collagen type I barrier interfaces underpins their growing potential as adaptable biological barriers in biomimetic microfluidic devices.

Table 1 highlights the similarities and differences between synthetic (engineered, crosslinked, or composite) and biological (native or reconstituted) collagen type I barrier interfaces used in microfluidic and OoC systems. Emphasis is placed on their biochemical cues, mechanical and transport properties, biodegradability, biomimicry, and applications, based on recent literature.

### 3.3. Fabrication Techniques of Collagen Type I Barrier Interfaces

Fabrication strategies for collagen type I barrier interfaces in biomimetic microfluidic devices encompass injection molding [54], electrospinning [19,36], in situ gelation [20,55], gel casting [7,56], (photo)lithography [57], and 3D bioprinting approaches [58,59], among others.

Injection molding becomes attractive when larger batches are needed for laboratory testing and performance validation. Unlike hot embossing—which can replicate standard lithographic features within certain bounds—microfluidic designs typically must be adapted for injection molding. In practice, this means adding draft angles to enable demolding (i.e., avoiding vertical sidewalls and undercuts) and keeping part thickness as uniform as possible to prevent filling defects during molding. The trade-off is cost: injection molding demands specialized, high-end equipment and dedicated molds, both of which can be expensive [54].

Shuxuan Jin and colleagues [57] developed a microfluidic platform that recapitulates the glioma immune microenvironment by co-culturing glioma cells and macrophages within a 3D matrix. The device comprised an upper PDMS layer bonded to a glass substrate, with the PDMS component fabricated via conventional soft lithography. In this setting, glioma cell spheroids exhibited markedly greater invasiveness in the presence of macrophages. Moreover, exposure to tumor cells shifted macrophage polarization from an M0 state toward an M2, tumor-supportive phenotype.

**Figure 1:**
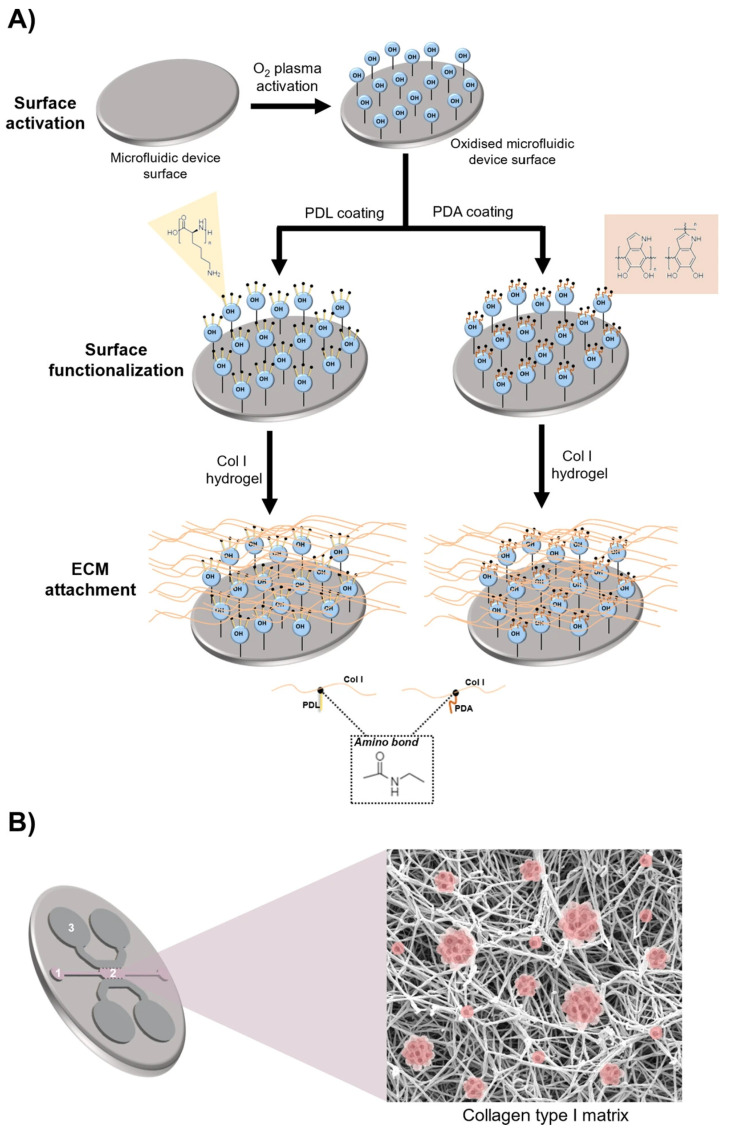
Experimental set up: (**A**) Activation and functionalization of PDMS microfluidic devices for long-term 3D culture. PDMS microfluidic devices were functionalized first by O_2_ plasma surface activation, followed by coating with PDL or PDA; (**B**) Schematic of the microfluidic device: single pancreatic cancer cells embedded in a collagen type I hydrogel are introduced through the loading port (1) into the central chamber of the device (2). Through the reservoirs (3), the culture medium is introduced. The isolated cells self-organize three-dimensionally, interacting with the matrix and generating tumor spheroids from single cells. Reproduced with permission from [60].

Hernández-Hatibi et al. [60] fabricated a PDMS microfluidic device using standard soft lithography. Hydrogels were prepared by combining rat-tail collagen type I with high glucose Dulbecco’s Modified Eagle Medium (DMEM). Pancreatic cancer cells were suspended as single cells in the collagen mixture, introduced into the central chamber through the loading ports (Figure 1), and allowed to polymerize for 20 min at 37 °C. After gelation, the constructs were hydrated via the reservoir ports (Figure 1) with high-glucose DMEM. The study evaluated two surface treatments—poly-D-lysine (PDL) and polydopamine (PDA)—to improve adhesion of collagen-based 3D matrices to PDMS. PDA coatings were notably effective, markedly strengthening the attachment of collagen type I hydrogels and enabling long-term culture of pancreatic cancer spheroids that exert substantial contractile forces on the surrounding matrix. By stabilizing matrix–device adhesion, PDA-coated chips support prolonged spheroid growth, prevent gel delamination, and preserve sustained cell-ECM interactions—features that together yield a more physiologically faithful platform for mechanistic and translational studies. More broadly, microfluidic 3D tumor models provide a closer approximation to in vivo tumor progression and may, in turn, inform improved diagnostic and therapeutic strategies for these difficult malignancies.

**Figure 2:**
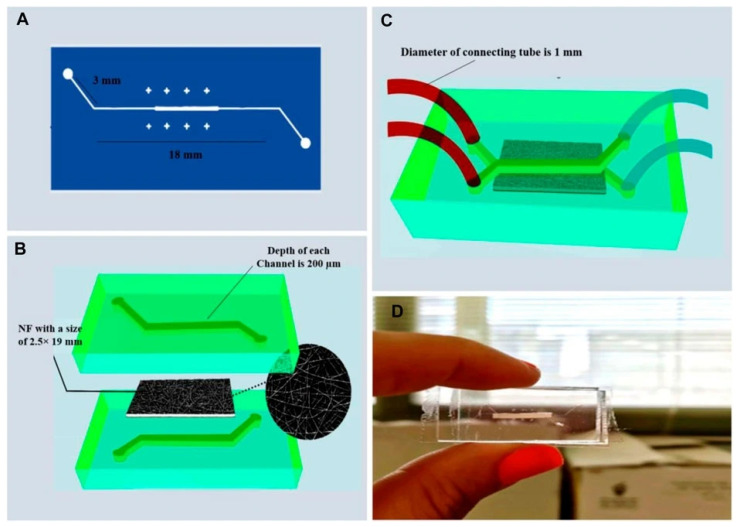
The MFC is based on collagen/PET/PDMS membrane: (**A**) A mask to pattern a photoresist; (**B**) PDMS chip casting with NF membrane (the porous nanofiber membrane is placed between the upper and the lower PDMS-based microchannels); (**C**,**D**) Schematic and real image of the PDMS microfluidic device. Reproduced with permission from [19].

Eslam et al. [19] developed collagen-modified PDMS/PET nanofiber barrier interfaces via two-nozzle electrospinning, followed by chemical crosslinking, which were then integrated into soft-lithography PDMS microfluidic chips for studying cell–nanofiber interactions (Figure 2).

Physical and chemical characterizations demonstrated that the cross-linked nanofibers, exhibiting a surface roughness of approximately 0.3 µm, possessed suitable hydrophilicity and biocompatibility. During the cell culture studies, they also proved to be appropriate scaffolds, showing low cytotoxicity and supporting cellular growth.

**Figure 3:**
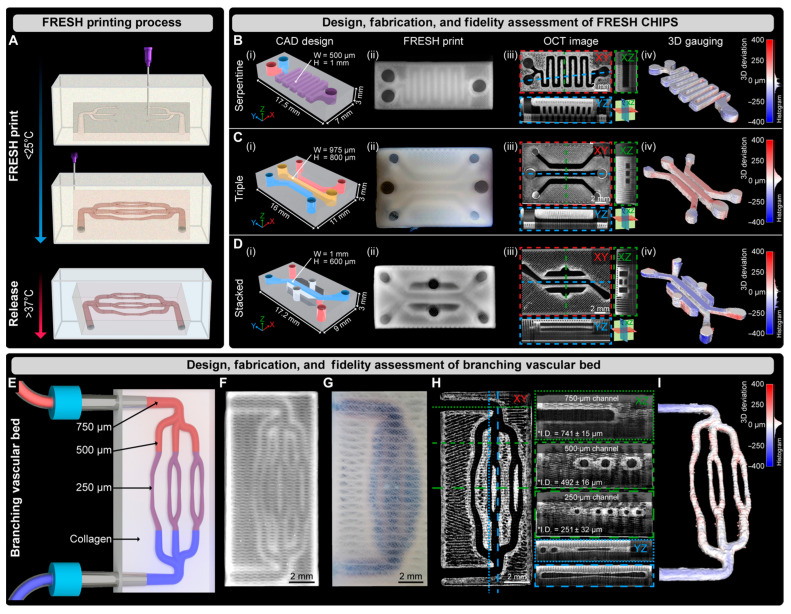
Fabrication, 3D gauging, and perfusion of FRESH-printed CHIPS: (**A**) Schematic of the FRESH bioprinting and release process. (**B**–**D**) CAD design (i); FRESH printing using a collagen bioink (ii); volumetric OCT imaging revealing high-resolution control over collagen extrusion and negative space resulting in patent lumens (dark regions) (iii); and 3D gauging of the negative space luminal region with histogram of the over and under print error for quantitative fidelity assessment of serpentine, triple, and stacked channel internal network designs. (**E**) A multi-scale vascular bed design with open lumens from 1 mm to 250 μm. (**F**) Multi-scale vascular bed FRESH printed from collagen I. (**G**) Perfusion of blue dye through the multi-scale vascular scaffold. (**H**) Volumetric OCT imaging and cross-sectional analysis demonstrating lumen patency and circular fidelity of the internal fluidic network. (**I**) 3D gauging of the multi-scale vascular lumen volume reveals high-fidelity printing with average deviations < 11 μm. Reproduced with permission from [58].

Bioprinting innovations have also emerged, with Shiwarski et al. [58] introducing collagen-based high-resolution internally perfusable scaffolds (CHIPSs) (Figure 3). Thus, ECM components and cells were 3D-printed into CHIPSs, which were subsequently integrated with a vascular and perfusion OoC reactor (VAPOR), resulting in a comprehensive tissue engineering platform.

They optimized the freeform reversible embedding of suspended hydrogels (FRESH) bioprinting technique to enable one-step fabrication of diverse CHIPS architectures. These constructs exhibited size-dependent permeability to perfused molecules, thereby supporting cell viability and migration within the surrounding scaffold.

Composite and hybrid barrier interfaces are increasingly used to enhance mechanical stability, adhesion, and handling properties of collagen type I barrier interfaces in microfluidic settings. In this respect, the PET/PDMS/collagen nanofiber barrier interface reported in study of Eslam et al. [19] is a clear example: blending collagen with PET and PDMS improved structural robustness and allowed for bonding to device layers (via oxygen plasma). It was thus demonstrated that microchannels with increased surface roughness (≈0.3 µm) exhibited a non-uniform shear rate distribution, and the flow rate significantly influenced both shear rate and velocity.

Surface immobilization or surface modification of device materials is also a key strategy. Thus, González-Lana et al. [17] reported on the modification of COP microfluidic devices via polyacrylic acid photografting (PAA-PG) or other treatments to allow stable collagen hydrogel adhesion and avoid device collapse in long-term culture.

In situ gelation (injecting or forming collagen gel directly inside channels) remains a promising route, especially for creating continuous ECM layers or lumen-lining in microdevices. Bioprinting (beyond CHIPSs) and in situ assembly permit embedding cells during gelation; these methods allow for controlled geometry and perfusable channels. The CHIPSs system demonstrates that perfusable channels can be printed into collagen type I bioink, allowing molecule diffusion from the lumen into surrounding ECM and supporting endothelial network formation, fluid flow, and cell migration [58].

Micro-molding, gel casting, and droplet/microgel techniques have been used in many barrier interface fabrication contexts, though fewer reports are purely collagen type I barrier interfaces in microfluidic devices. For example, microfluidic droplet generators have been used to make collagen–alginate microgels for single-cell encapsulation, which shows how gel casting/droplet techniques can embed collagen in microfluidic settings [61]. In their review of OoC barrier interface fabrication methods, Corral-Nájera et al. [7] identified gel casting and hydrogel mold filling as standard strategies for producing protein/ECM-based barrier interfaces, with tunable parameters such as thickness, porosity, and crosslinking.

A microfluidic human liver-on-chip for modeling drug-induced liver injury (DILI) was engineered using a combination of 3D stereolithography printing, computer numerical control (CNC) milling technology, and molding [62]. The goal was to create a 3D liver model that reflects key aspects of human hepatic physiology and pathophysiology; a sinusoid-like architecture, sustained cell viability with preserved phenotypes, and measurable liver specific functions. In principle, the resulting platform also supports the controlled induction and study of distinct disease phenotypes, providing a versatile testbed for DILI and related hepatotoxic processes.

**Figure 4:**
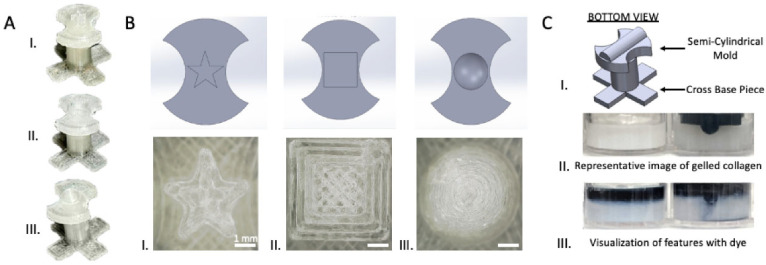
3D printed molds: (**A**) Images of fully assembled printed molds in (I) star-shaped, (II) square and (III) round well shapes. (**B**) Top view of mold shapes to demonstrate ability to create different architectures in hydrogels. (I) Star-shaped CAD model and digital microscope image of star-shaped printed mold. (II) Square-well CAD model and digital microscope image of square-well printed mold. (III) Round-well CAD model and digital microscope image of round-well printed mold. (**C**) (I) SolidWorks2021 model of fully assembled open-channel mold, showing bottom view of cross-base piece (bottom) and open channel (top). (II) Side view of gelled collagen without mold (left image) and with 3D-printed mold before mold removal (right image). (III) Side view of gelled collagen after mold has been removed, revealing open-channel architecture, vs. gelled collagen with no molded shape. Scale bars: 1 mm. Reproduced with permission from [48].

O’Brien et al. [48] introduced an open microfluidic cell-culture platform fabricated from 3D-printed molds (Figure 4).

Compared with conventional closed microfluidic systems, the approach is easier to manufacture, requires only minimal specialized equipment, and offers improved access for sampling and manipulation. The molds can be used to cast a range of hydrogels into tissue-mimetic architectures, including endothelialized channels that recapitulate blood-vessel-like structures using human umbilical vein endothelial cells (HUVECs).

Pereira Guimaraes et al. [21] fabricated a tubule-on-a-chip by combining 3D printing with soft molding. The device supports co-culture of renal proximal tubule epithelial cells (RPTECs) and (HUVECs). It was shown that the RPTECs: HUVECs co-culture adhered efficiently within 30 min inside microchannels pre-treated with plasma, 3-aminopropyltriethoxysilane, and collagen type I—substantially shortening the time needed before initiating medium perfusion. Taken together, the platform offers a practical route for assessing nephrotoxicity of drug candidates and probing drug–cell interactions in a controlled co-culture, while also helping to reduce reliance on animal models and support safer, more ethical pharmaceutical development.

### 3.4. Modifications and Crosslinking Strategies of Collagen Type I Barrier Interfaces

Collagen type I barrier interfaces used in microfluidic devices often require modifications or crosslinking to meet mechanical, permeability, and biochemical robustness demands. Among chemical crosslinking agents, genipin has been shown to increase stiffness with relatively low cytotoxicity. For example, Ishihara Seiichiro et al. [63] demonstrated that collagen gels crosslinked with genipin at increasing concentrations (0 to 10 mM) can produce Young’s moduli in the range ~0.03 to ~12.5 kPa, with minimal toxicity to lung cancer cells (H1299) and mesenchymal stromal cells. In addition, Santiviparat et al. [64] employed genipin crosslinking in combination with 3D bioprinting to advance cell-tissue engineering, enabling the precise layering of cell-containing matrices while maintaining low cytotoxicity. These findings suggest that genipin is a viable strategy for tuning stiffness in barrier interfaces where flow or shear demands are modest (e.g., microfluidic chips mimicking soft tissues).

Another common crosslinking strategy of collagen is 1-ethyl-3-(3-dimethylaminopropyl)carbodiimide with N-hydroxysuccinimide (EDC/NHS). These zero-length crosslinkers can improve mechanical strength, reduce swelling, and enhance enzymatic stability. For instance, Sionkowska et al. [65] studied fish collagen films crosslinked with EDC and EDC/NHS under different conditions. They found that immersion crosslinked films had significantly lower swelling and increased durability (in phosphate-buffered saline, PBS), changes in Young’s modulus and tensile strength, and altered surface roughness and hydrophilicity depending on whether NHS was included. While this study is not in a microfluidic device per se, its results are directly relevant for barrier interfaces: lower swelling, improved tensile strength, and predictable degradation are all properties needed when barrier interfaces are under perfusion or fluid pressure.

GTA remains another strong chemical crosslinker, although with trade-offs in cytotoxicity. In a microfluidic context, De Angelis et al. [66] designed a multilane device for human enzyme immobilization (FMO3). A microfluidic immobilized enzyme reactor was developed comprising four separate serpentine channels, in which FMO3 and its two common polymorphic variants (V257M and E158K) were covalently immobilized using GTA crosslinking on a poly-L-lysine (PLL) coating. For proof-of-concept validation, the platform was characterized with respect to channel coating (collagen vs. PLL, both compatible with GTA-mediated crosslinking), available surface area for immobilization, and applied flow rate. The highest product yield was achieved at a flow rate of 10 mL min^−1^ on PLL-coated serpentines with the largest surface area (90 mm^2^). Owing to its ease of surface functionalization, as well as high enzyme retention and activity, the lysine-based crosslinking method was identified as the most effective strategy for immobilizing human FMO3 on this multi-channel microfluidic platform. This illustrate that while GTA can provide strong covalent crosslinks desirable for long-term barrier integrity, its use in microfluidic devices must be optimized to avoid damaging nearby cells or barrier interfaces.

UV (photo-) crosslinking is a physical/chemical strategy that has become more utilized in recent years. A recent study by Zhang et al. [30] on UV-cured atelocollagen barrier interfaces showed that the wet-state compression modulus (E_c_) and swelling ratio (SR) were significantly affected by the UV-cured network architecture, leading up to a three-fold reduction in SR and about two-fold increase in E_c_ in the sequentially functionalized, compared to the single-functionalized, samples. The study’s demonstrated structure–property relationships confirmed the key role played by the molecular architecture of covalently crosslinked collagen, aimed towards long-lasting resorbable barrier interfaces for predictable guided bone regeneration therapy. Thus, this kind of UV-crosslinking can be useful in microfluidic barrier interfaces because it allows spatial control of crosslinking (e.g., mask-based, or UV exposure through covers), possibly enabling patterning of stiffness or barrier strength across an interface.

Hybrid crosslinking strategies also show promise. Bilateral or double crosslinking of EDC with GTA is one example: in this novel bilateral treatment strategy—employing GTA on the stromal side and EDC on the basement barrier interface side of the decellularized human amniotic (dHAM)—an effective balance was achieved between mechanical reinforcement and biocompatibility. Specifically, GTA improved the mechanical properties of the stromal surface, while EDC preserved the cytocompatibility of the basement barrier interface side. By evaluating the handling characteristics and biological performance of this cross-linked barrier interface, the study presents a promising approach to repurposing a well-established biomaterial through a dual-modification process, potentially advancing its application in wound healing and regenerative medicine [67]. Although such approaches have not yet been demonstrated in microfluidic barrier interfaces, they suggest that implementing gradient or asymmetric crosslinking strategies could offer a balanced solution—enhancing mechanical strength while preserving biocompatibility.

To summarize, Table 2 presents practical criteria for possible selection of a collagen type I crosslinking strategy in biomimetic microfluidic systems.

## 4. Integration of Collagen Type I Barrier Interfaces in Microfluidic Devices

### 4.1. Methods of Integrating Collagen into Microfluidic Platforms

#### 4.1.1. Sandwiching Pre-Formed Collagen Barrier Interfaces Between PDMS or Other Polymer Layers

Recent studies revisited the “classical” two-layer chip with a collagen type I barrier interface mechanically clamped or bonded between microchannels, but with upgraded barrier interface formats (electrospun or vitrified collagen and collagen-functionalized synthetics) and cleaner bonding workflows. Thus, Eslam et al. [19] built a PET/PDMS microfluidic chip where an electrospun PET nanofiber sheet was surface-modified with collagen type I and sandwiched between plasma-activated materials based on PDMS and PET. It was demonstrated that the plasma exposure plus the nanofiber roughness yielded leak-free bonding. It was concluded that the as-fabricated device could be used as a rapid test for drug screening, permeability, cell viability measurements, and disease modeling.

This “collagen-on-scaffold” strategy also appears in lung and nanoparticle-toxicology chips. Kim et al. [68] developed in their study a kidney-on-chip (KoC) platform to evaluate nephrotoxicity. The device featured apical and basolateral chambers separated by a PET membrane coated with collagen type I, supporting co-culture. Under flow conditions, robust cell barrier integrity was reported. The model was demonstrated to maintain epithelial barrier integrity and enabled functional assessments, including glucose reabsorption.

Manufacturing routes for collagen-dominant inserts are diversifying. Thus, the work of Cenhrang K et al. [69] presents a microfluidic–transwell hybrid device, in which an electrospun collagen scaffold is sandwiched between two laser-cut Teflon membranes to provide a barrier for epithelial culture and functional assays.

The design of the collagen type I scaffold underscores how intermediate collagen barrier interfaces can be integrated between rigid supports (the Teflon membrane), i.e., functioning like a reinforced “sandwich” barrier. In this architecture, the collagen scaffold was shown to act as a pathway for trans-epithelial transport.

#### 4.1.2. In Situ Formation and Patterning Strategies for Collagen Type I

In biomimetic microfluidic platforms, the in situ formation and spatial patterning of collagen type I barrier interfaces represent key strategies for replicating native ECM structures and their functional roles in selective transport and cell–matrix interactions. These approaches enable the localized generation of continuous, physiologically relevant interfaces with minimal alteration of microchannel geometry, while allowing fine control over spatial gradients in mechanical stiffness or biochemical composition. Such tunability is essential for mimicking tissue-specific barrier properties, including permeability and cellular adhesion. Nevertheless, achieving reproducible in situ collagen fibrillogenesis under microfluidic constraints (e.g., confined geometry, convective flows, limited diffusion of neutralizing agents or crosslinkers) remains technically challenging.

**Figure 5:**
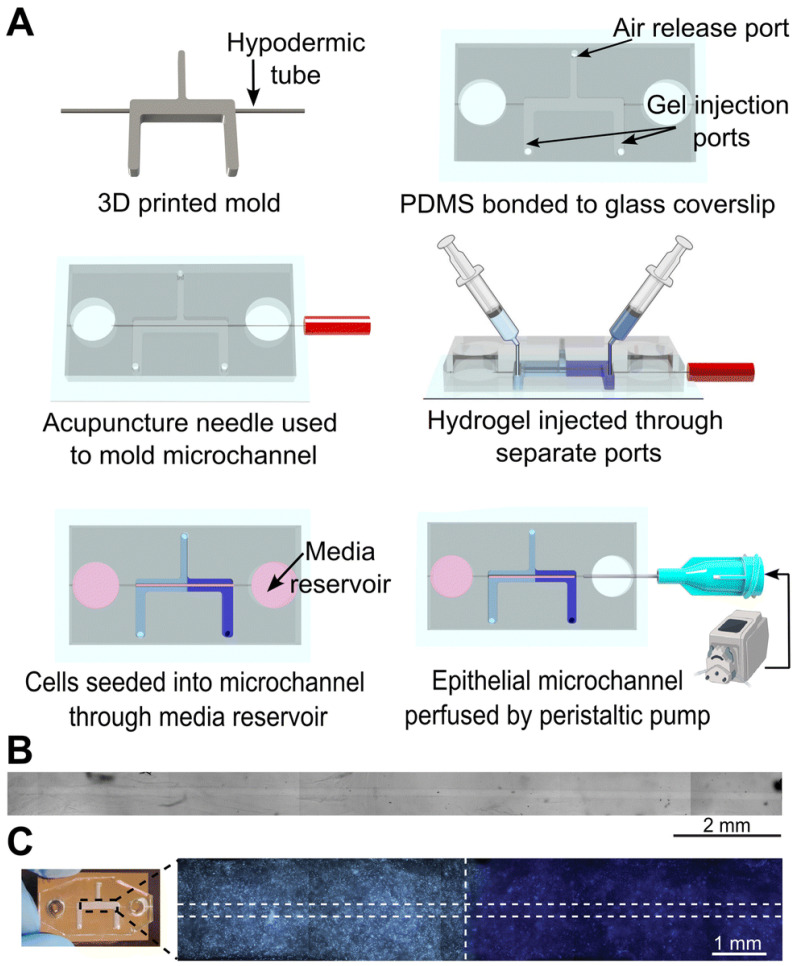
Fabrication and perfusion of a continuous central microchannel within a regionally patterned microchannel device: (**A**) Fabrication process for device; (**B**) Brightfield image of a hydrogel microchannel within a device before addition of cells; (**C**) Fluorescent labeling with microspheres of patterned collagen hydrogel device. Horizontal dashed lines indicate edges of microchannel. Vertical dashed line indicate interface between the two regions. Reproduced with permission from [70].

Chernokal et al. [70] report a microphysiological system in which distinct hydrogel zones (with different compositions) are serially patterned around a continuous central microchannel by orthogonal injection of prepolymer solutions and partial annealing (10–30 s apart) to allow interfaces to anneal while preserving zonal identity (their own properties). In their scheme, they inject hydrogel prepolymer (e.g., collagen-based) segments sequentially, allowing partial polymerization before the next zone is injected, thus achieving a continuous but spatially heterogeneous hydrogel barrier around the channel (Figure 5).

This is a clever variant of in situ gelation during microchannel fabrication, enabling longitudinal patterning of collagen-based ECM without interrupting the central lumen. They verify that the gel–gel interfaces do not hinder diffusion of solutes or disrupt perfusion, and they show region-specific epithelial sprouting from the lumen into the surrounding ECM, demonstrating functional coupling.

Middelkamp et al. [71] developed and systematically set up a 3D blood vessel-on-chip (VoC) with embedded (lipid-laden) macrophages, using sequential cell seeding in viscous finger patterned (VFP) collagen hydrogels. They then endothelialized the lumen and embedded macrophages in the surrounding matrix to test coagulation under whole-blood perfusion.

The obtained results demonstrated a significantly higher fibrin coverage in the channels containing embedded macrophages, suggesting a proinflammatory effect exerted by these macrophages, and consequently, an enhanced endothelial response.

**Figure 6:**
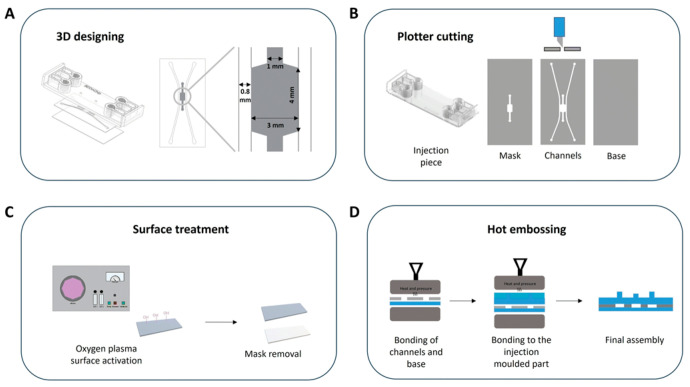
Fabrication process: (**A**) Design of the device—3D model and 2D channel layer with details of the central chamber area; (**B**) Material selection and cutting: the device is made of olefin copolymers and plotter cutting is used to achieve the desired geometries. The design of the central channel is used to obtain the mask and the central and lateral channels; (**C**) Surface treatment: oxygen plasma surface activation process using the mask with the central chamber geometry; (**D**) Thermocompression bonding: firstly, the channel layer is bonded to the base; finally, channels are bonded to the injection piece. Reproduced with permission from [72].

Olaizola-Rodrigo et al. [72], addressed a pervasive limitation in hydrogel microfluidics: confinement strategies that rely on micropillars can distort local shear fields and restrict direct cell–cell interactions at interfaces. To overcome this, the authors introduced a new microfluidic fabrication route for pillar-free thermoplastic chips and validated it in a biological-relevant setting (Figure 6). Notably, the method supports a broad range of chamber geometries with tunable, well-controlled shear stress distributions by establishing diffusion profiles uninterrupted by pillars.

Using a plasma-based patterning/activation workflow in thermoplastic substrates, they generate “abutment-free” boundaries that confine hydrogels without physical posts. Collagen type I (4 mg mL^−1^, rat tail) was loaded and gelled in situ. The gel remained confined within the central chamber under both static and perfused conditions, while exhibiting time-dependent diffusion consistent across the tested designs. Building on this platform, the team assembled a BBB-on-a-chip and quantified barrier function in the pillar-free device. They observed formation of a continuous endothelial barrier that recapitulates key features of the brain’s neurovascular interface. Overall, the results highlight the potential of this microdevice to support functional 3D BBB models suitable for evaluating candidate therapies for central nervous system disorders.

O’Brien et al. [48] introduced an open-microfluidic approach in which semi-cylindrical channels are molded directly into collagen type I (2–4 mg mL^−1^) using simple 3D-printed molds; after collagen gelled (15 min at RT, followed by 1 h at 37 °C), the posts were removed to reveal the architecture, and HUVECs were seeded to generate vessel mimics. The molded channels match their theoretical dimensions across sizes down to ~400 µm and across collagen densities, enabling robust, geometry-faithful constructs. Ussing these vessels, the team modeled mild hypoxia (16% O_2_) vs. normoxia (20% O_2_), observing reduced viability (87.2% vs. 95.5%) and lower CD31 coverage (24.9% vs. 32.3%) under hypoxia. The method is inexpensive and accessible with benchtop resin or filament printers. Finally, while the approach yields open channels rather than fully closed lumens, the authors note advantages for sampling and monitoring—features that can be desirable for barrier-interface studies where one side intentionally remains exposed.

**Figure 7:**
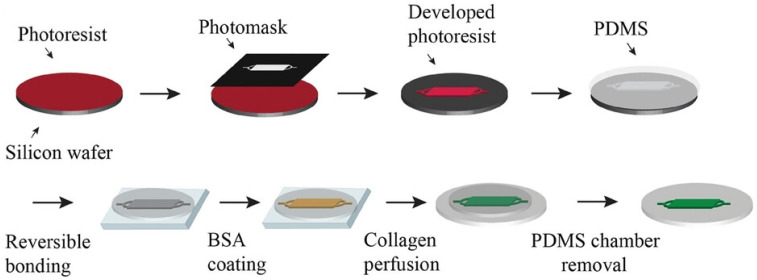
Concept, design, and characterization of the microfluidic platform: schematic of the fabrication process of the microfluidic devices (**top** row) and collagen micropatterns (**bottom** row). Reproduced in part with permission from [73].

Giacomini et al. [73], introduced a simple piggy-back microfluidic platform that lays down aligned, fibrillar collagen type I directly on standard culture substrates. In practice, a PDMS device carrying engineered micropillars is briefly placed on a glass-bottom dish, a neutralized collagen solution is flowed through the pillar network, and the collagen is allowed to polymerize. The top piece is then lifted away, leaving behind pre-patterned, anisotropic collagen tracks on the substrate surface (Figure 7).

These flow-guided patterns generate robust fiber alignment (especially with 50 μm pillar spacings), and tenocytes grown on them adopt the expected elongated morphology while maintaining a tenogenic program. Crucially for interface engineering, the authors demonstrated material transferability: by imprinting the aligned collagen topography into polystyrene, they decouple biochemistry from structure and show that the fibrillar anisotropy itself drives tenocyte shape, while collagen biochemistry primarily tunes marker expression. This means the same directional ECM cues can be ported onto common thermoplastics. Because the device is only temporarily mounted to pattern the substrate—and then removed—the method does not produce a sealed microchannel. Instead, it yields an open, pre-patterned ECM interface that is easy to seed, image, and sample. For barrier-interface studies where matrix anisotropy at an abutting surface (e.g., stromal fibers meeting an endothelium) can modulate mechanics and paracrine exchange, this openness is a practical advantage rather than a drawback.

**Figure 8:**
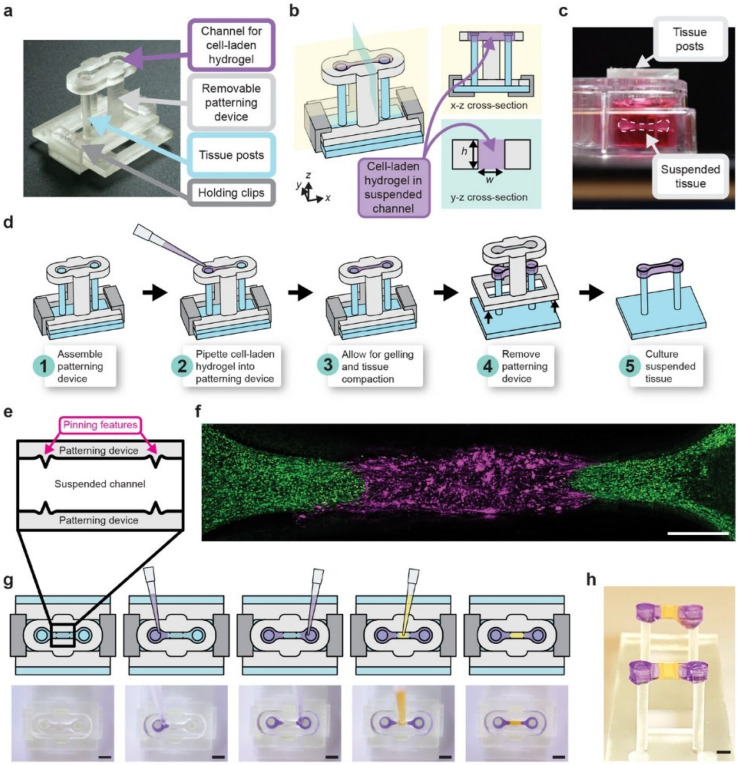
Workflow of generating single and multi-region suspended tissues using the STOMP platform: (**a**) Image of the STOMP platform, which includes a removable patterning device containing an open channel that interfaces with a pair of vertical posts. The patterning device is held in place to the base of the posts with holding clips. (**b**) Schematic of the STOMP platform: a cell-laden hydrogel is pipetted into the open channel, where it flows via surface-tension driven forces across the open channel and anchors onto the suspended posts, thus generating a free-standing suspended tissue. (**c**) Side view of the resulting suspended tissue cultured in a 24-well plate. (**d**) Workflow of patterning a tissue composed of a single region, where the composition is the same across the tissue. (**e**) Top-down view of the capillary pinning features along the open channel that are used to pin the fluid front. (**f**) Fluorescent image of patterned 3T3 mouse fibroblast cells laden in a fibrin hydrogel using STOMP. The outer region of 3T3 cells was dyed by CellTracker Green (green) and was pipetted first. The middle region of 3T3 cells was dyed by CellTracker Red (magenta). Scale bar: 500 µm. (**g**) Workflow of patterning tissues comprising three distinct regions. Scale bars: 2 mm. (**h**) Side view image of multi-region agarose suspended hydrogel construct. Scale bar: 2 mm. Reproduced with permission from [74].

Haack et al. [74] introduced suspended tissue open microfluidic patterning (STOMP), an open-microfluidic, capillary-pinning strategy for building multi-region, freely suspended tissues. By machining simple “pinning” features into a removable patterning device, one hydrogel was pipetted after another so that the advancing fronts arrested at predefined edges, then intentionally met to form clean, contiguous borders (Figure 8).

In practice this lets them combine native ECM—explicitly including collagen type I—within a single construct, so a healthy–fibrotic boundary or a bone–ligament junction can be created on demand and then interrogated mechanically. Using this format, changes in cardiac tissue contractility were quantified across a patterned fibrotic domain and read out force generation in periodontal models with mineralized and soft regions. This demonstrated how emergent behavior depends on the interface itself. The authors reported contact angles for collagen type I (5 mg mL^−1^) on 1% bovine serum albumin (BSA)-treated 3D-printed resin of 30° and connected the measurements to pinning thresholds. The quantitative link between measured contact angle and pinning success is rare in tissue-patterning papers and makes it straightforward to select pin shapes/angles or swap materials when laying down discrete collagen subdomains of different densities or compositions within a single construct.

#### 4.1.3. Immobilization of Collagen onto Chemically or Physically Treated Surfaces

As microfluidic platforms have become the default scaffolds for barrier models, surface conditioning has moved from a “setup detail” to a first-order design variable. Collagen type I, in particular, is indispensable for turning otherwise inert channel walls into biologically competent interfaces; but on native PDMS, cyclic olefin copolymer (COC)/COP, or titanium and other substrates, simple physisorption rarely survives shear, contraction, or long runs. Recent work has therefore leaned on covalent chemistry (e.g., silanes in combination with bifunctional crosslinkers, PDA catechol, acrylic photo-grafts) and on physical activation (oxygen/argon plasma) to immobilize collagen in ways that hold up under flow and cell-mediated remodeling.

To anchor collagen type I gels against contraction in injection-molded COP-based microfluidic devices, González-Lana and co-workers [17] systematically compared oxygen plasma, 3-aminopropyltriethoxysilane (APTES) → GTA silanization, and PAA-PG, with a PDL/GTA route as an adsorption-plus-covalent benchmark. Collagen was loaded into a 2 mm-wide central chamber flanked by perfusable side channels. The covalently tethered, carboxyl-rich PAA graft on COP not only mitigated collapse and detachment but also enabled long-term, diffusion-limited cultures. Collectively, the reported results position PAA-PG as the most robust immobilization strategy for collagen type I on COP, providing a reliable foundation for barrier-interface studies that depend on sustained matrix integrity under load and perfusion.

Hernández-Hatibi et al. [60] illustrate a pragmatic route to immobilize collagen type I within PDMS microchannels by combining a standard physical activation step with a single aqueous PDA coating. Plasma-oxidized PDMS was first “primed” with PDA, a catechol-bearing film that forms by dopamine auto-oxidation and readily couples to nucleophilic groups on biomolecules. Collagen type I hydrogels were next loaded and seeded with pancreatic ductal adenocarcinoma cells (PDACs) into the PDA-treated chambers. In direct comparison with PDL, PDA created a markedly more tenacious collagen–PDMS interface: collagen gels remained confined and intact for up to 11 days even as tumor spheroids generated substantial contractile forces that otherwise delaminated gels from PDL-coated channels. Notably, deposition time did not control adhesion within the tested window. They subsequently fine-tuned PDA concentration and rinsing to balance matrix anchorage against the mechanical constraints sensed by cells. It was thus demonstrated that, a single-step PDA interlayer transformed otherwise fragile collagen coatings into robust, flow-resistant barriers on physically activated PDMS—an uncomplicated surface-chemistry solution that secures long-term collagen immobilization without sacrificing biological performance.

Kefallinou and colleagues [75] offered a clean, device-centric recipe for anchoring collagen type I on PDMS: a single air-plasma activation, performed during chip sealing, followed by prompt collagen deposition. This minimalist sequence converts the inherently hydrophobic elastomer into a uniformly wettable substrate on which collagen forms a continuous, superhydrophilic layer that resists the rapid detachment typical of purely adsorbed films. Contact-angle analyses and seven-day stability tests showed that plasma-induced oxidation, when “captured” by timely collagen coating, markedly slows hydrophobic recovery and preserves interfacial integrity. Most importantly, the authors validate the approach inside 3D PDMS microchambers, where mesenchymal stem cells expand to full confluence over five days with improved homogeneity relative to native, unmodified devices. In short, a single plasma step—followed directly by collagen type I layering—yields a stable, cell-supportive interface that materially improves collagen retention and downstream culture performance in real microfluidic hardware.

**Figure 9:**
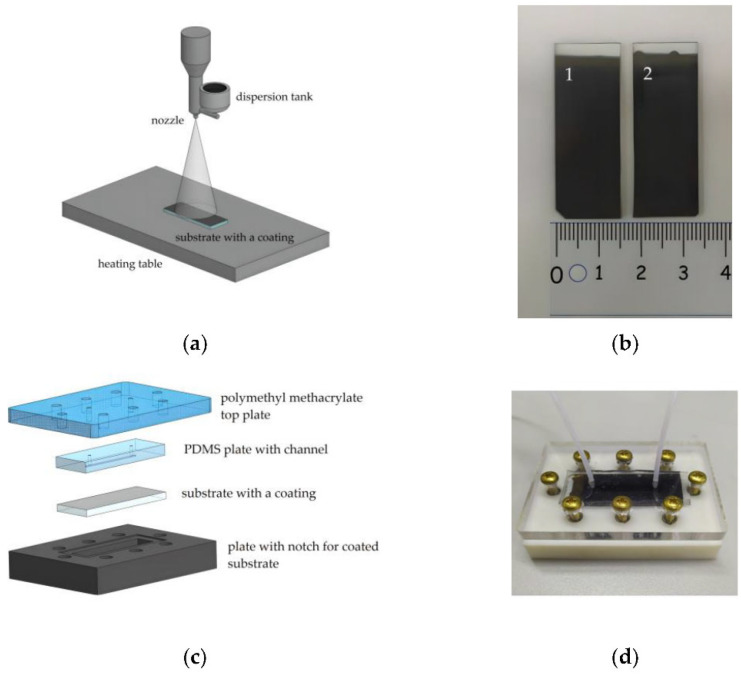
(**a**) The scheme of aerosol layer-by-layer deposition; (**b**) substrates with applied coatings based on collagen/c-MWCNTs (1) and collagen/c-MWCNTs/GTA (2); (**c**) scheme and (**d**) photo of the fabricated microfluidic chip. Reproduced with permission from [76].

Popovich et al. [76] approached collagen immobilization by embedding multi-walled carbon nanotubes (MWCNTs) within a collagen matrix and, in a second variant, chemically crosslinking the collagen network with GTA (Figure 9).

Within this study the authors evaluated how firmly these hybrid collagen layers remained attached when challenged by shear in a purpose-built, reversible microfluidic chip—letting them swap samples and re-run flow without destroying the device. Under phosphate-buffered flow, profilometry showed a clear benefit from crosslinking: the GTA-treated composite degraded roughly half as much as the non-crosslinked film after 3.5 h (δ ≈ 2.75% vs. 5.5%). In parallel hemocompatibility assays with albumin flow, Raman and Energy-dispersive spectroscopy (EDS)/Scanning electron microscopy (SEM) readouts indicated less protein adsorption on both composites than on Ti controls, with the non-crosslinked MWCNTs/collagen showing the lowest detectable albumin. Together, these experiments speak directly to a central practical concern—whether collagen coatings stay put under shear—and illustrate that mild chemical crosslinking can substantially toughen collagen-based interfaces while maintaining favorable blood-facing behavior.

Slepičková Kasálková et al. [77] used a rigorous physical-chemistry route to immobilize collagen type I on device-grade PDMS microgrooves. Firstly, linear microstructures were imprinted by photolithography, activated the patterned elastomer with argon plasma, and then laid down a collagen type I coating. It was demonstrated that a hydrophobic pristine PDMS (water contact angle ~109°) became strongly hydrophilic (~24°) after plasma, and—critically—settled to ~54° once collagen was deposited. On these collagen-functionalized grooves, C2C12 myoblasts were shown to adhere, spread, and align robustly along the pattern direction. Mechanistically, it was argued that plasma introduced reactive sites that coupled with amino groups in collagen, creating stronger interfacial bonds and reducing the risk of delamination. In short, the study nailed the materials side of immobilization: argon-plasma activation provides a durable anchor for subsequent collagen layers on microstructured PDMS, yielding stable, cytocompatible interfaces suited for device-level applications.

#### 4.1.4. Development of Hybrid Devices Combining Collagen with Synthetic Polymers

Hybrid microfluidic platforms—where a soft, fibrillar collagen type I barrier is physically or chemically integrated with a synthetic polymer chip—have matured noticeably in the past years. What distinguishes these systems is not only the choice of polymer (PDMS, COP/COC, PET) but the interface engineering that prevents delamination, leakage, or gel collapse during perfusion. According to the recent literature, most groups converge on one of three routes: (i) covalent or graft-mediated immobilization of collagen to otherwise hydrophobic thermoplastics or silicones; (ii) insertion of a collagen-bearing membrane (often a collagen-modified PET nanofiber mat) between PDMS layers; (iii) in-channel patterning of collagen hydrogels whose stability is enhanced by local chemistry or composite additives.

Recent work by González-Lana et al. [17] demonstrated a practical route to hybrid devices that integrate collagen type I hydrogels with low-gas-permeable thermoplastics. Using injection-molded COP chips featuring a central gel compartment flanked by perfused side channels, the authors systematically compared surface chemistries (i.e., APTES silanization, PDL/GA amination, and PAA-PG) for immobilizing collagen under strongly contractile conditions (human cardiac fibroblasts at ~10^7^–2 × 10^7^ cells/mL). Across both standard (1.2 mg/mL) and stiffer (4 mg/mL) collagen formulations, PAA-PG consistently delayed or prevented gel collapse over multi-day culture while maintaining cell viability; by contrast, APTES performed poorly and PDL/GTA was intermediate, approaching PAA-PG only at higher collagen concentration. Importantly, the COP substrate’s low gas permeability enabled stable oxygen and nutrient gradients, allowing formation of density-dependent necrotic cores without loss of gel integrity—an ischemia-like scenario that is difficult to sustain on PDMS-centric platforms. Conceptually, these findings show that with an appropriate graft chemistry, thermoplastic microdevices can host long-lived, contraction-resistant collagen barriers, broadening the material palette for long-term barrier and gradient assays beyond the PDMS default.

Eslam et al. [19] presented in their work a pragmatic hybrid platform that integrates a collagen-bearing nanofiber membrane into a standard two-layer PDMS chip. The membrane—electrospun from collagen-modified PET with PDMS—was designed explicitly to emulate a basement-membrane-like barrier and, crucially, to process like a polymer part. The reported results illustrate a straightforward “drop-in” route to add a collagen-rich barrier to PDMS microfluidics, combining the manufacturability of PET/PDMS with the biological cues of collagen while making the hydrodynamic consequences of the hybrid interface explicit.

Several groups refined PDMS surface activation to anchor collagen as a durable inner lining under flow. Kefallinou et al. [75] reported that brief plasma activation of PDMS, followed by collagen type I immobilization, converted an otherwise bioinert elastomer into a robust, cell-forward hybrid interface. In sealed microfluidic chambers, air-plasma applied at the bonding step enabled a single-stage route to collagen lining that remained highly wettable for a week and supported dense, uniform mesenchymal stem cells (MSCs) layers for at least five days, outperforming non-functionalized controls and avoiding the uneven coverage typical of simple physisorption. Together, these data argue that stability of the collagen–PDMS junction during culture is the key benefit of the plasma-enabled approach. Complementing the microchannel work, argon-plasma treatment of microstructured PDMS provided chemically active sites that bonded collagen type I and resisted peel-off, producing cytocompatible, guidance-promoting surfaces for myoblasts with clear improvements in adhesion, proliferation, and alignment [77]. The authors document the practical aspects of plasma modification and collagen coating on patterned PDMS—details that translate readily to both open and enclosed PDMS architectures. Collectively, these experiments amount to a reliable recipe for hybrid collagen–PDMS devices: activate by plasma, immobilize collagen, and the coating withstands days of handling and medium exchanges without the detachment commonly seen after adsorption alone.

**Figure 10:**
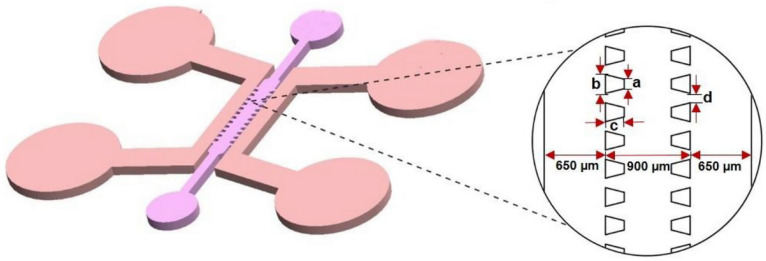
The schematic of the microfluidic system: the device is composed of two lateral media channels and one interposed gel channel. Reproduced with permission from [15].

Ghobadi et al. [15] built a pillar-confined PDMS–glass microfluidic device in which a central, collagen type I gel channel communicated with lateral perfusion channels across trapezoidal capillary-burst posts (Figure 10).

They numerically optimized the post geometry and gap (≤100 µm) so the gel would fill cleanly and retain its microchannel shape during handling. Into this collagen matrix the authors dispersed BG NPs. Conceptually, the study exemplifies hybridization at a collagen–synthetic barrier: a natural ECM gel integrated with a synthetic microchip is reinforced by an inorganic nanophase to improve handling and stability while preserving cellular compatibility—an approach directly aligned with emerging hybrid collagen–polymer device strategies.

Alcaide et al. [78] created 3D cylindrical micro-vessels by seeding endothelial cells into collagen type I gels cast inside a standard PDMS microfluidic chip, and then asked a pointed barrier question: does enriching the collagen scaffold with laminin and hyaluronan tighten the endothelium? The evidence says yes. When laminin/HA (MatriMix) was incorporated into the collagen matrix surrounding the lumen, ZO-1 localized at junctions and diffusive permeability decreased, indicating a more robust barrier. Notably, the improvement arose from chemistry rather than architecture—the device format and overall physical properties stayed comparable—showing that collagen’s function as the barrier-supporting scaffold can be tuned biochemically to yield “tighter” vessels without changing chip geometry. This is a clear example of hybrid collagen–polymer microdevices in which subtle ECM compositional edits at the collagen–PDMS interface enhance endothelial integrity and function.

To further clarify which strategies are most suitable for specific applications, a trade-off table (Table 3) summarizes ease of fabrication, biological fidelity, mechanical robustness, and cost for integrating collagen into microfluidic platforms.

### 4.2. Design Considerations

#### 4.2.1. Thickness of Barrier Interfaces

In microfluidic devices intended to mimic biological barriers or allow intercellular communication across compartments, the thickness of a collagen type I barrier interface is a critical design parameter. It mediates a trade-off among mechanical stability, diffusive transport, and the physical proximity needed for effective paracrine or juxtacrine signaling between co-cultured cell types. Too thick a barrier interface may impose excessive diffusion resistance or spatial segregation; too thin a barrier interface may lack structural integrity, delaminate, or fail to support handling and cell seeding.

It is important to note that a review of recent literature on collagen or ECM-based barrier interfaces in microfluidic and OoC systems reveals a notable scarcity of direct measurements or reporting of collagen type I barrier interface thickness. Nevertheless, the following studies provide some informative data points.

In their study, Eslam et al. [19] described a hybrid nanofiber barrier interface composed of electrospun PET and collagen is embedded in a PDMS microfluidic device to mimic basement barrier interface-like behavior for co-culturing cells on opposite sides of the barrier interface. The barrier interface is a nanofiber mat, so its “thickness” is not presented with a single thick bulk value, but rather as a porous network. The good cell viability and adhesion they observed suggests that the thin nanofiber structure is sufficient to support cell contact and mass exchange. Their use of numerical simulation (COMSOL MULTIPHYSICS 5.5) to assess flow fields and shear distribution further indicates the sensitivity of microfluidic barrier interfaces to the topography and thickness (or effective height) of the barrier layer.

Another promising advance is the work of Zhang et al. [56], which describes ultrathin collagen sheets that are still mechanically handleable, pushing toward collagen barrier interfaces with thicknesses of a few microns. This strategy employs templated collagen sheets as substrates with strong potential for bottom-up bio-fabrication of load-bearing biomaterials for soft tissue repair. By mimicking the non-linear tensile response while retaining tensile strength, it enables hierarchical material design, particularly relevant to cardiovascular tissue engineering.

A hybrid fluorescent nanofibrous barrier interface composed of poly(ε-caprolactone) (PCL) and collagen was fabricated by Kanabekova et al. [79] for integration into a microfluidic device constructed from COP and PDMS. The resulting PCL–collagen barrier interface exhibited a thickness of approximately 10 μm, demonstrated excellent biocompatibility both within and outside the microfluidic environment, and possessed mechanical properties comparable to those of the native basement barrier interface. The implementation of PCL–collagen barrier interfaces in lung-on-a-chip platforms has been proposed as a promising strategy to enhance the in vitro representation of the alveolar–capillary barrier. By leveraging the structural and biological advantages of PCL–collagen barrier interfaces alongside the capabilities of microfluidics and real-time imaging of cell–barrier interface–fluid interactions, this approach holds potential to deepen the understanding of pulmonary pathophysiology, accelerate drug discovery processes, and ultimately contribute to improved therapeutic outcomes.

In the architecture presented by Cenhrang et al. [69], the collagen scaffold functioned as a compliance buffer, necessitating a thickness that was sufficiently low to minimize diffusion resistance, yet adequate to preserve mechanical coherence between layers. The study demonstrated that this design enabled selective molecular transport while maintaining the integrity of the cell monolayer. These findings support the concept that, in microfluidic devices, collagen interfaces of intermediate thickness—typically on the order of tens of microns—can effectively mediate diffusive exchange while simultaneously supporting cell adhesion and maintaining barrier tightness.

#### 4.2.2. Porosity and Pore Size

When integrating collagen type I barrier interfaces as biological barrier layers in microfluidic devices, the barrier interface’s microstructure—especially porosity and pore radius/size—becomes an important parameter. The porosity impacts convective or diffusive transport of solutes, the hydraulic permeability, as well as cell migration or transmigration (if desired). However, in the recent literature, relatively few works report fully characterized collagen type I barrier interfaces with explicit pore metrics in microfluidic settings.

Eslam et al. [19] present in their study a composite nanofibrous barrier interface composed of collagen-modified PET and PDMS nanofibers, inserted between PDMS microchannels to mimic a basement-interface–like barrier. The authors measured via Brunauer, Emmett, and Teller (BET) a total pore size volume and an average pore diameter, and have shown that it decreased from 0.06 cm^3^/g and 12.25 nm to 0.01 cm^3^/g and 3.41 nm after crosslinking. It was concluded that the fabricated device holds significant potential for use in rapid drug screening, permeability assessment, cell viability evaluation, and in vitro disease modeling.

Choi et al. [80] integrated collagen hydrogels (type I) into a microfluidic angiogenesis assay, and examined how nonenzymatic glycation (via D-Ribose) affects collagen microstructure, endothelial sprouting, and cell migration. They incubated 3 mg/mL collagen with ribose (0, 100, 200, 300 mM) for five days to introduce glycation crosslinks, then polymerized the gel in situ within the microfluidic chip. The authors do not report explicit pore diameters, but the qualitative SEM and distribution of fiber spacing provide a semi-quantitative sense of the network density. Functionally, they showed that endothelial sprouts invade more deeply in the more crosslinked (i.e., more open) conditions (200–300 mM glycation), with larger lumen sizes, suggesting that sparser structure may facilitate migration.

#### 4.2.3. Mechanical Stability

In microfluidic devices intended to impose continuous flow, shear stress, or trans-barrier interface pressure gradients, mechanical integrity and adhesion (to supports) of the collagen type I barrier interface become critical. Without sufficient mechanical stability, barrier interfaces may deform, delaminate, rupture, or sag, compromising barrier function, fluid partitioning, or cell culture integrity. One could mention here some of the key strategies to enhance mechanical performance: (i) chemical or physical crosslinking of collagen; (ii) composite designs (e.g., reinforcing fibers or support meshes); (iii) optimized bonding to device substrates (e.g., plasma activation, covalent coupling).

Eslam et al. [19] reported on the fabrication of hybrid nanofiber barrier interfaces by two-nozzle electrospinning of collagen-modified PET and PDMS, assembled between upper and lower microfluidic channels. They performed tensile testing on the barrier interfaces before and after chemical crosslinking, in the dry state. They have demonstrated that the crosslinked barrier interfaces exhibited greater elongation (i.e., improved ductility) relative to the non-crosslinked counterparts. The authors also assessed adhesion/bonding: after oxygen plasma activation, the fibers were bonded between PDMS and PET layers. The device reportedly sustained 24 h of perfusion at 10 µL/min without any unwanted leak. Even though the values of shear were modest, the fact that the barrier interface survived 24 h of flow suggests a minimal threshold of mechanical robustness in the authors’ design.

Ghobadi et al. [15] embed collagen type I hydrogels loaded with varying percentages of BG NPs (1%, 2%, 3% *w*/*v*) inside a microfluidic chip, and report rheological measurements of the hydrogels: storage modulus (G′) and loss modulus (G″). In the absence of flow, their collagen-only hydrogel (no BG NPs) exhibited a value for G′ ≈ 64.7 Pa, whereas the collagen + 3% BG NPs variant increased G′ to ~761 Pa (i.e., ~12 × stiffer) under small-strain rheometry. Even though the authors did not perform mechanical testing under microfluidic flows (i.e., no burst pressure, delamination tests, or tensile testing), they used the rheological values to argue that incorporation of BG NPs enhanced mechanical stability enough to resist collapse or leakage during static culture. It is known that rheological shear moduli are not directly equivalent to tensile strength or barrier interface integrity under shear or pressure; however, they give a baseline for comparative stiffness. Moreover, in their microfluidic culture, the authors claimed no leakage of collagen gel into neighboring media channels and stable confinement for days of culture, indirectly supporting mechanical integrity under perfusion conditions. To conclude, one could mention that this approach, i.e., composite reinforcement by NPs, is relevant and may analogize to composite barrier interface strategies.

In another recent work relevant to microfluidic collagen thin films, Zhang et al. [56] describes a microfluidic wet-spinning/templating process to produce ultrathin, templated collagen sheets with hierarchical organization. The authors report mechanical properties of their collagen type I barrier interfaces (wet state) including tensile strength and modulus. Thus, as strain increased, the progressive straightening of collagen fibrils resulted in a 62-fold increase in the elastic modulus. This non-linear mechanical response closely resembles the tensile behavior observed in native cardiovascular tissues, a feature that has not yet been successfully replicated in ultrathin collagen sheets. The barrier interfaces are described as “handleable,” indicating that they exhibit sufficient mechanical integrity to resist tearing or buckling during manipulation and integration into microfluidic assemblies. Although the study did not extensively evaluate their performance under dynamic conditions—such as integration into chip modules or exposure to prolonged shear flow (e.g., delamination under fluidic stress)—the mechanical characterization data suggest a promising direction toward the development of barrier interfaces with robustness adequate for various fluidic applications.

#### 4.2.4. Bonding with Device Materials

The successful integration of collagen type I as a functional barrier interface in microfluidic devices necessitates the establishment of stable and reproducible bonding with device materials, most commonly PDMS, thermoplastics, or glass. This bonding is critical not only for the mechanical integrity and leak-free operation of the assembled system but also for maintaining the physiological relevance of the collagen interface under flow conditions. However, achieving robust adhesion between collagen-based hydrogels or films and synthetic substrates remains technically challenging due to differences in surface chemistry, interfacial energy, and the susceptibility of collagen to hydration-dependent structural changes. Several strategies have been proposed to improve bonding, including surface activation, chemical functionalization, interfacial layering, and in situ polymerization techniques. These approaches must be evaluated in terms of their compatibility with biological components, preservation of collagen’s structural hierarchy, and long-term mechanical and fluidic performance.

Eslam et al. [19] report that the collagen-functionalized nanofibrous barrier interface was incorporated into a PDMS microfluidic chip by oxygen plasma activation of the PDMS and PET surfaces, allowing bonding of the nanofiber barrier interface between the upper and lower microchannels. After bonding, they ran 24 h perfusion at 10 µL/min through the microfluidic device with no mention of leakage or delamination, suggesting that the bond was sufficient to maintain integrity under that flow regime. This paper is a particularly relevant example from the literature, as it presents a practical and reproducible bonding method—plasma activation—that ensures flow-compatible integrity over extended periods.

Another important study is the work of González-Lana et al. [17] who compared various treatments such as physisorption, amination with PLL, covalent silanization, and covalent PAA-PG for immobilizing collagen gels (or coatings) in COP/PDMS-based microfluidic devices. They observed that physically adsorbed collagen coatings tend to detach under medium incubation or contractile stress, while covalent immobilization, e.g., via PAA-PG or PDL + GTA, improved retention of the hydrogel structure over days. It was thus reported that PDL + GTA crosslinking delays hydrogel contraction/detachment from PDMS/COP surfaces, and that the PAA-PG method (i.e., covalent grafting) outperforms PDL in long-term stability.

#### 4.2.5. Biocompatibility

Collagen type I, as a principal structural protein in many connective tissues, offers intrinsic bioactivity: its integrin-binding motifs (e.g., GFOGER, DGEA) promote cell adhesion, spreading, and survival without the need (or with minimal need) for supplementary coating. In contrast, synthetic barrier interfaces typically require adsorption or grafting of ECM proteins (e.g., fibronectin, laminin) to support cell anchorage. Thus, collagen-based barrier interfaces are particularly appealing as barrier scaffolds in microfluidic devices, offering a more native cell–matrix interface.

However, in microfluidic systems the barrier interface (or hydrogel barrier) is subject to perfusion shear, limited nutrient access, and possible delamination or remodeling. Therefore, demonstrating biocompatibility under device-relevant conditions (flow, confined geometry, long culture) is essential.

Evidence from Hernández-Hatibi et al. [32] underscores that collagen type I barriers are broadly biocompatible with PDACs epithelium-like lines while still revealing cell-type-specific sensitivities to matrix mechanics. Across collagen type I concentrations spanning 2.5–6 mg/mL, classical subtype cells with well (Capan-2) and moderately (BxPC-3) differentiated morphology and expression of epithelial markers consistently self-organized into viable 3D spheroids, indicating that the barrier material supports epithelial tumor cell adhesion, survival, and collective remodeling without overt cytotoxicity. In contrast, mesenchymal-biased lines required denser gels to achieve stable 3D assemblies, highlighting that biocompatibility of collagen interfaces should be interpreted functionally—as the capacity to sustain physiologically appropriate growth modes—rather than as mere absence of toxicity. Using population-level readouts (area over 10 days) and the 90th-percentile (p90) metric to temper median-based bias from newly nucleated micro-spheroids, the study showed that collagen concentration modulates spheroid size distributions in a line-specific manner. BxPC-3 formed fewer and more uniform spheroids in softer matrices (2.5 mg/mL), whereas higher collagen (4–6 mg/mL) favored larger, more variable structures with elevated p90—compatible with robust proliferation and remodeling within the collagen barrier. Capan-2 remained comparatively homogeneous across matrices and maintained high p90 values—most notably at 6 mg/mL—suggesting that increased fibrillar density does not impair, and may even favor, the formation of sizable epithelial spheroids [32]. Taken together, these behaviors indicate that collagen type I barriers can be tuned to remain biocompatible across PDAC phenotypes while preserving discriminatory power: epithelial-like cells thrive across a wide range of collagen densities, whereas mesenchymal-like cells demand stiffer, tighter networks before adopting stable 3D growth. For microfluidic designs, this argues for selecting collagen type I barrier concentrations that both (i) maintain cell health and epithelial organization in classical lines and (ii) avoid under-constraining mesenchymal lines that would otherwise default to two dimensional (2D) outgrowth—thereby ensuring that “biocompatible” interfaces also elicit biologically faithful morphogenesis.

In the study of Ghobadi et al. [15], the authors embed L929 fibroblasts in collagen type I hydrogels (3.0 mg/mL) with varying loads of BG NPs: 1%, 2%, 3% *w*/*v*), and inoculate them into a microfluidic device. The 3-(4,5-dimethylthiazol-2-yl)-2,5-diphenyltetrazolium bromide (MTT) cytotoxicity assays on BG NPs and live/dead staining on L929 cells inside the collagen–BG NPs constructs within the device were conducted. The results indicated high viability of fibroblasts across the tested BG NPs concentrations; the sample with 3% BG NPs (i.e., collagen3-BG NPs3) was selected as optimal. Because the collagen is the principal ECM matrix, the results support that collagen-based matrices remain biocompatible and supportive of cell survival in a microfluidic architecture under perfusable (though low-flow) conditions.

In another study, Eslam et al. [19] engineered a dual-channel PDMS microfluidic device incorporating a nanofibrous barrier interface composed of PET electrospun fibers coated with collagen type I. This biofunctionalized barrier interface was inserted between two microchannels, enabling co-culture of HUVECs and C6 glial cells on opposite sides of the barrier. Under continuous perfusion at 10 µL/min for 24 h, fluorescence microscopy revealed confluent and morphologically appropriate cell attachment on both surfaces of the barrier interfaces. The authors attributed the observed biocompatibility and adhesion stability under shear conditions to the presence of collagen, which improved cellular affinity compared to uncoated synthetic barriers. These results underscore the functional advantage of collagen modification in promoting stable barrier integration and dual-cell support in perfusable microfluidic architectures.

Another important work linking device-anchoring strategies (to better immobilize collagen in microdevices) with preservation of cell viability over extended culture is the study of González-Lana et al. [17]. It addresses viability of cells embedded in collagen matrices inside microfluidic or microdevice geometries, and how surface anchoring treatments affect long-term retention of the collagen and cell viability. They compared various surface treatments, e.g., PDL in combination with GTA, APTES silanization, polyacrylic acid photografting) on COP-based microfluidic devices to promote the immobilization of collagen as a 3D matrix protein. They quantified cell viability by calcein/propidium iodide staining after 13 days: all treatments showed no statistically significant reduction in viability compared to controls (i.e., the treatments did not introduce cytotoxicity) within their experimental error bounds. They also visually tracked hydrogel area retention (i.e., how well the collagen remained in place) over time. It was concluded that the PAA-PG treatment best prevented detachment of the collagen matrix over days.

The work of O’Brien et al. [48] used microfluidic or “microchannel-like” hydrogel embedding of vasculature-mimetic systems and monitored viability under normoxic (20% O_2_) and hypoxic (16% O_2_) conditions. The hydrogel matrix acts a barrier interface relevant to how collagen (or ECM) matrices support cell viability in microfluidic settings. Their results demonstrated that under hypoxic vs. normoxic conditions, viability declines by ~8.3%. Since the matrix is part of the device-integrated environment (i.e., hydrated gel combined with microfluidic perfusion), this work is of particular interest because is illustrative of how ECMs in microenvironments support or constrain viability under stress.

#### 4.2.6. Co-Culture Spatial Arrangements

Beyond thickness, porosity, and mechanics, the way different cell types are positioned relative to a collagen type I barrier interface strongly shapes mass transport, morphogenesis, crosstalk latency, and the readouts one can trust (transendothelial/epithelial electrical resistance—TEER, tracer flux, and cytokine gradients). In this respect, three spatial motifs may recur—each with distinct engineering variables and translational benefit.

##### Apical–Basal (Surface) Seeding onto a Collagen-Bounded Compartment

In an alveolus-on-chip [81], fibroblasts embedded in a collagen type I gel underlay an apically seeded airway epithelium at an air–liquid interface (ALI), while an opposing endothelial channel fosters the development of a more physiologically relevant environment. Practically, this configuration (i) isolates barrier integrity from stromal remodeling, (ii) makes ALI straightforward, and (iii) links readouts to collagen-mediated signal delay.

In the microfluidic stomach-on-a-chip work from Ferreira et al. [82], primary gastric fibroblasts were embedded within a collagen type I gel (0.5–3 mg/mL), while seeding gastric epithelial cells apically. The results of the study showed how fibroblast-mediated collagen remodeling proceeded under flow without compromising epithelial barrier formation and function, and it highlighted density-dependent gel stability important for long experiments. This configuration is directly relevant when collagen type I serves as the lamina propria analogue beneath an epithelial barrier.

##### Lateral (Side-by-Side) Interfaces Across a Collagen Type I Gel Lane

In their study, Russell et al. [83] cultured an endothelial monolayer along a perfused channel and positioned a second tissue (human induced pluripotent stem cell-derived brain spheroids) laterally across a collagen type I gel lane (7–8 mg/mL). They quantified how collagen type I density tuned neurite extension and integration with the vascular network while maintaining perfusability. Because diffusion and convection occur orthogonally across the collagen type I lane, this geometry is well-suited for studying directional transport, gradient maintenance, and guided angiogenic sprouting from an endothelium into collagen type I.

##### Embedded Tri- or Multiculture in a Single Collagen Type I Compartment with Luminal Seeding

Du et al. [84] presented in their work a vascularized bile duct-on-a-chip where mesenchymal cells were embedded in a collagen gel forming the interstitial barrier between a perfused biliary channel and a perfused vascular channel. The configuration enabled immune-cell transmigration across the endothelial barrier toward the epithelium under defined cytokine stimulation, illustrating how an embedded collagen matrix can function simultaneously as a structural barrier and as a signaling conduit across two perfused compartments.

#### 4.2.7. Oxygen Gradient Management

##### Air–Liquid Interface Delivery

Airway chips, where oxygen was supplied apically by air, while nutrients were delivered from the basal/peripheral side, with collagen type I providing the interstitial barrier, were recently reported [85]. In a collagen tubular airway-on-chip, Gao and his collaborators maintained a confluent epithelium for two weeks under continuous air perfusion through a collagen tube while medium was presented externally—an explicitly ALI interface configuration that stabilized epithelial function and highlighted how oxygen was supplied independently of liquid flow. The work justifies ALI set-ups when epithelial maturation is the priority and note the need to anchor collagen structures to avoid compaction during prolonged aeration.

##### ALI-Dominated Oxygen Delivery

Licciardello et al. [81] developed a tri-culture alveolus-on-chip comprising an electrospun PCL–gelatin membrane that separated apical and basolateral chambers. A fibroblast-laden collagen type I hydrogel resided on the apical side beneath the epithelial monolayer, while endothelial cells lined the basolateral side. After epithelial confluence, the apical compartment was exposed to ALI, so oxygenation of the epithelium was governed primarily by the air phase, whereas basolateral perfusion supported endothelial viability and nutrient exchange. This architecture is best cited as an ALI-first configuration that still leverages a collagen type I interstitium for stromal–epithelial crosstalk.

##### Explicit Oxygen Profiling

To anchor design choices in direct measurements, a tumor-microenvironment device was used to quantify oxygen tension inside a 3D matrix (a fibrinogen-based hydrogel primarily composed of fibrinogen, with added collagen type I), while co-culturing endothelial cells and tumor spheroids. By embedding oxygen-responsive reporters and tuning hydrogel properties, Chiang et al. [86] uncovered key features of tumor–endothelium crosstalk, showing in particular how endothelial cells modulate hypoxia. This platform delivers the first quantitative measurements of oxygen dynamics in these settings and offers a flexible framework for probing spatial and environmental stress factors.

#### 4.2.8. Sampling and Monitoring Port Placement

Open-top and reservoir-layer chips use elevated, off-axis media wells to generate gravity-driven perfusion while keeping the culture lanes free of local suction jets. In an open-top, barrier-free vascularization chip, Alma Yrjänäinen et al. [87] positioned reservoirs away from the 3D compartments and validated flow fields (experiment and modeling) across several inlet configurations. The design supported long, stable perfusions and preserved lumenized microvessels—conditions that are notoriously sensitive to sampling-induced recirculation. This study is referenced as a concrete template when locating sampling ports for collagen type I gel lanes.

In a platform for vascularized organoids-on-chip [9], secretions were collected downstream while perfusion through the endothelialized path remained uninterrupted. The team explicitly schematized glucose-stimulated insulin secretion sampling without touching the encapsulation trap, and demonstrated that anastomosed microvessels stayed perfusable during repeated assays. This study clearly justifies the successful placing of sampling tees after the collagen-bounded compartment.

For continuous monitoring, two complementary device-level demonstrations are summarized. The first one reports on a gut-on-chip with multiple flexible TEER electrodes laminated along the channel walls [88]. The authors mapped barrier formation at four locations and discussed how tape-mounted thin-film electrodes prevented pockets and sealing issues that can compromise flows. It was shown that the normalized TEER value for the obtained data stabilized at ~75 Ω·cm^2^. The second study [89] relies on an off-stoichiometry thiolene-based microphysiological system with in situ oxygen and pH sensors distributed before/after the treatment zone, enabling paired measurements under both stop-flow and continuous-flow conditions. Importantly, the integrated sensors did not degrade viability, and the two-position layout illustrated how to read gradients across a test segment without introducing sampling side-streams into the culture lane.

#### 4.2.9. Manipulating Collagen Type I Architectural Design

To keep the review practical, this subsection looks at how collagen type I can be deliberately shaped on microfluidic channel surfaces to guide barrier formation and function. Recent reports show that straightforward choices—surface activation and grafting chemistries, electrospun or patterned collagen interfaces, and microfabrication routes that align fibers or cast tubular ECM—allow investigators to tune fibril order, porosity, wettability, and curvature with surprising precision. These architectural variables then modulate epithelial and endothelial behaviors that matter for barrier performance—adhesion, planar polarity, junction maturation—and, ultimately, TEER and solute permeability. The aim here was therefore to extract the few controllable levers that consistently deliver better, more stable collagen–cell interfaces in perfused devices.

(i) Surface activation and grafting on thermoplastics to immobilize collagen hydrogels (planar channel walls): in COP chips, oxygen plasma, APTES in combination with GTA, and especially PAA-PG provided progressively stronger anchoring of collagen type I gels against contractile cell traction, preventing gel detachment/collapse over multi-day culture [17]. The same work quantifies hydrogel retention vs. time and shows viability is preserved, offering a practical recipe for long-term barrier assays performed on thermoplastics.

(ii) Electrospun collagen-modified membranes bonded into PDMS channels to control nano-/micro-topography: a collagen-modified PET/PDMS composite membrane, produced by two-nozzle electrospinning and oxygen-plasma bonding, was integrated between parallel channels. Membrane cross-linking and roughness systematically shifted pore volume, hydrophilicity, and shear profiles, thereby controlling cell adhesion and flow exposure at the barrier interface [19]—i.e., a bottom-up route to define the microtexture that cells “feel”.

(iii) Flow-guided collagen fiber alignment inside microchannels (anisotropy on demand): recent microfluidic platforms show that channel geometry and flow can intrinsically align collagen type I fibers during in situ polymerization, yielding spatially programmable anisotropy [90]. This approach lets investigators pattern aligned vs. random regions and superimpose chemokine gradients to parse how architecture steers epithelial morphogenesis and protrusion dynamics.

(iv) Microfluidically aligned fibrillar tracks patterned onto culture substrates (piggyback device): a user-friendly “piggyback” microfluidic device printed fibrillar collagen type I micropatterns with tunable alignment on the substrate beneath cells. It was shown that alignment alone lengthens cell morphology and modulates lineage marker expression, cleanly linking fibril order to phenotype [73] (the same principle applying also to epithelial alignment and barrier orientation).

(v) Pre-formed collagen type I tubules as the lumen-facing barrier (tubular channel architecture): collagen tubes were demonstrated as the primary channel structure; in a high-throughput gut-on-chip in which Caco-2 tubules were formed against collagen type I and used for TEER-coupled toxicology, the collagen geometry (tube vs. flat sheet) was the lever that set epithelial organization and functional readouts [91].

(vi) 3D-printed devices with integrated electrospun collagen scaffolds (hybrid fabrication for barrier inserts): additively manufactured, Transwell-style microfluidics that sandwich electrospun collagen scaffolds between laser-cut membranes give precise control of scaffold thickness and fiber diameter while retaining perfusion and standard area normalization [69]—useful when one wants Transwell-like handling with microfluidic control.

(vii) Fully bioprinted, perfusable collagen-based microfluidics (architectural control by print path): high-resolution collagen bio-printing produces internally perfusable, ECM-only microfluidic networks (“CHIPS”), where channel caliber, curvature, and wall thickness are defined by the print tool path and gelation conditions [58]. This offers a bottom-up way to fix barrier curvature and wall mechanics while remaining in a collagen type I matrix.

Together, these reports show that collagen type I architecture is controllable at two levels: surface chemistry (e.g., plasma/silanization/photografting to immobilize and shape gels on channel walls) and microfabrication/topology (electrospinning, microfluidic alignment, bioprinting, and tubular casting) that set fibril order, porosity, and 3D geometry.

#### 4.2.10. Microfabrication Challenges

The integration of collagen type I barrier interfaces within microfluidic systems poses a series of microfabrication challenges that differ substantially from those encountered with synthetic polymers or inert substrates. While collagen offers inherent bioactivity and ECM-like properties essential for mimicking physiological microenvironments, its soft, hydrated, and structurally dynamic nature complicates reproducible device assembly and long-term stability. Notably, collagen barrier interfaces are highly susceptible to dimensional changes upon hydration, leading to unpredictable swelling that can distort microscale architectures, alter flow paths, and compromise sealing fidelity. Furthermore, the relatively low interfacial adhesion strength between native collagen matrices and common microfluidic substrates (e.g., PDMS, glass, thermoplastics) increases the risk of delamination under fluidic shear or pressure gradients. These problems are exacerbated by batch-to-batch variability in collagen extraction and gelation behavior, which can hinder reproducibility in barrier interface thickness, porosity, and mechanical performance. Collectively, these limitations underscore the need for tailored fabrication strategies—such as controlled crosslinking, composite reinforcement, and optimized bonding protocols—to ensure consistent integration of collagen type I barrier interfaces in microfluidic architectures, while preserving their biological function.

Eslam et al. [19] conducted dye injection tests using blue water–soluble dye to evaluate the sealing performance of their nanofiber integrated microfluidic channels. Following oxygen plasma bonding, no leakage was observed for at least 10 min, indicating short-term sealing integrity of the device. The composite barrier interface, composed of collagen embedded with a PET/PDMS electrospun nanofiber matrix, likely contributed to structural integrity by reducing swelling and delamination tendencies often associated with pure collagen. However, due to the hybrid nature of the barrier interface, the individual swelling behavior of collagen may be obscured by the dimensional conferred by the synthetic polymer matrix. This highlights a key challenge in microfabrication: the trade-off between biological relevance and mechanical robustness in composite barrier structures.

In their recent study, Shiwarski et al. [58] addressed critical microfabrication challenges associated with 3D bioprinting of collagen-based scaffolds, particularly regarding lumen fidelity and structural integrity under perfusion. Employing optimized FRESH printing, the authors achieved high-resolution internal channels, with an average root-mean-square deviation of less than 11 µm from the computer-aided design models, as confirmed via volumetric optical coherence tomography-based gauging. These results highlight a reproducible fabrication approach for collagen microarchitectures. Furthermore, successful dye perfusion experiments demonstrated patent, mechanically stable lumens, indicating that the bio-printed collagen structures maintained integrity under flow conditions, with no apparent deformation or collapse within the tested perfusion regime.

**Figure 11:**
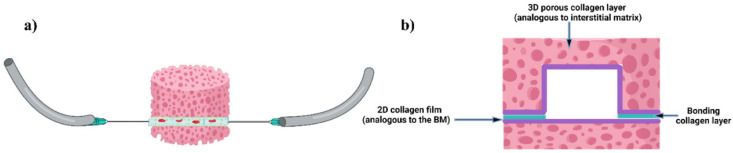
Schematic diagrams of the proposed collagen scaffold simulating 3D porous tissues with embedded microchannels that have inner-wall 2D membrane linings: (**a**) The final scaffold with an embedded microchannel connected to inlet and outlet tubing; (**b**) Close-up cross-sectional view of the microchannel with the 2D BM lining embedded inside the 3D porous IM, which was formed by bonding two collagen layers. Reproduced with permission from [92].

Fakhri et al. [92] present in their work a collagen-based scaffold integrating a thin 2D collagen film (designed as a basement membrane analogue) bonded to a 3D porous collagen bulk and containing enclosed microchannels fabricated by contact microprinting (Figure 11).

Dimensional fidelity was quantified by comparing channel geometry to a PDMS mold: the fabricated heights and perimeters are ~20% smaller than mold values (reflecting shrinkage during processing), while widths more closely match the target dimensions. This discrepancy was interpreted as evidence of the inherent limitations in reproducibility when using collagen scaffolds subject to collapse or densification during freeze-drying procedures. To assess structural integrity of the bonded interface under flow, they performed leakage tests across a range of volumetric flow rates. For scaffolds lined with the 2D collagen film, measured outflow equals inflow in all tested conditions (up to 10 mL/min), indicating no detectable convective leakage or delamination. In contrast, when the 2D film is omitted, the porous 3D collagen scaffold allows penetration of flow at low rates, demonstrating the key role of the dense film in sealing the interface. Collectively, these results illustrate two central microfabrication challenges in collagen-based vascular models: (i) the dimensional shrinkage and loss of fidelity during scaffold processing, especially in the height and perimeter directions; (ii) the need for robust bonding/lamination methods that resist delamination and leakage under physiologically relevant flows.

### 4.3. Computational Modelling and Machine-Learning Frameworks for Collagen Type I Barrier Interfaces

Collagen type I barrier interfaces in microfluidic devices sit at the junction between mechanics and transport. Cells remodel the matrix, flow compacts it, and both processes shift pore architecture and, in turn, solute permeability. Capturing this loop benefits from two complementary toolkits: physics-based finite-element analysis (FEA) and data-driven models that learn structure-function mappings from experiments.

#### 4.3.1. Finite-Element and Poro-Mechanical Analyses of Collagen Gels Under Physiologic Loading

To connect continuum modeling directly to barrier transport, recent work coupling compression rheology with FEA to extract permeability and poro-elastic responses in collagen-based networks was reported. Thus, Mollenkopf et al. [93] performed controlled compression experiments on collagen gels and used FEA to interpret stress relaxation and pore pressure fields, revealing strain-dependent densification regimes that govern fluid flux (Darcy permeability) in fibrillar matrices. Permeability decreased nonlinearly with successive compression, indicating that flow is controlled by network morphology. Clarifying how mechanical loading, microstructure, and fluid transport interact in biopolymer networks—especially collagen gels—will inform experiments designed to improve mass transport in native and engineered tissues.

Complementing this, Cacheux et al. [94] combined bespoke instrumentation with kinetic modeling to show pronounced tension–compression asymmetry in collagen gels: compression stiffens the network and reduces permeability, whereas these effects largely vanish after chemical cross-linking. The experimental–model pairing provides boundary conditions and constitutive trends (e.g., asymmetric permeability–strain relationships) that could be explicitly cited as inputs/validation targets for future FEA of collagen type I barrier interfaces in chips.

Another microfluidic device work where geometry-driven shear fields are verified experimentally and supported by computational flow simulations, which is directly relevant to barrier interface function under controlled shear, was reported by Olaizola-Rodrigo et al. [72]. The authors have developed a pillar-less microfluidic platform to allow direct contact between cells, recreate different geometries mimicking tissue structures, and generate specific shear stress profiles to obtain more accurate experimental models. Simulations were used to map shear distributions that were then realized experimentally—an approach pointed for coupling FEA-derived wall stresses with measured permeability/TEER in collagen-lined channels.

#### 4.3.2. Machine-Learning Models That Predict Barrier Permeability with Experimental Validation

The study of Huang et al. [95] developed a transformer/XGBoost classifier to classify BBB permeability with in vitro validation. The authors trained models on curated BBB datasets and then validated predictions in 3D human BBB spheroids (human brain microvascular endothelial cells, brain vascular pericytes, and astrocytes), showing good agreement between in silico (computational) predictions and measured permeation for a held-out compound set. The validation platform represents an experimental barrier system and directly demonstrates that machine learning-predicted permeability can anticipate measured transport across a living human barrier. Thus, this strategy can be ported to collagen type I microfluidic barriers by (a) generating paired permeability/TEER and confocal readouts under defined shear, (b) extracting morphological/junctional features, and (c) training models to predict permeability from structure–function descriptors—mirroring the logic of Huang et al. but with collagen type I matrices and chip-specific covariates (shear, matrix density, and cross-linking).

#### 4.3.3. Molecular-Dynamics Simulations to Link Self-Assembly to Mesoscale Barrier Interface Properties

At the molecular scale, new experimental–computational studies clarify how hydration and accessory collagen domains tune collagen type I assembly kinetics and the resulting network morphology–parameters that ultimately control barrier porosity. Giubertoni et al. [26] combine isotopic substitution experiments with coarse-grained molecular dynamics (MD) to show that water composition modulates intermolecular interactions, accelerating nucleation and altering the final network. This MD-supported mechanism is crucial to justify why collagen gelation conditions (including solvent composition) should be part of any predictive barrier interface model.

Chowdhury et al. [96] paired kinetic measurements of collagen type I fibrillogenesis with atomistic MD to demonstrate that specific isoforms of collagen α1(XI) N-terminal domains alter activation energies and growth rates during assembly. This supports treating isoform identity as a model parameter and, by extension, as barrier permeability in collagen type I interfaces.

## 5. Functional Roles of Collagen Type I Barrier Interfaces in Microfluidics

### 5.1. Barrier Function

The primary functional role of a collagen type I-based barrier interface in microfluidic systems is to mediate selective transport—facilitating the diffusion of small solutes, nutrients, and signaling molecules, while restricting the passage of larger molecules, cells, or particles. In such microfluidic architectures, barrier performance is typically evaluated through permeability or diffusion coefficients, and in more advanced analyses, by reflection (or rejection) coefficients. Given that the barrier interfaces in many microfluidic devices are composite in nature—comprising both collagen matrices and cellular layers—disentangling the specific contribution of the collagen matrix from that of the cellular component remains a methodological challenge. Nevertheless, recent experimental studies have provided valuable insights into the barrier function of collagen-based matrices and have proposed improved methodologies for quantifying selective permeability in these systems.

In the study by Eslam et al. [19] the barrier function of the collagen/PET/PDMS nanofiber membrane was investigated primarily through structural, physicochemical, and biological assessments rather than direct permeability assays. The nanofiber membrane demonstrated increased fiber diameter (from 391 nm to 660 nm), enhanced surface roughness (from 66.9 nm to 296.7 nm), and improved hydrophilicity (contact angle reduction from 105° to 58.8°). These modifications are critical for barrier formation, as they were demonstrated to promote stronger cellular attachment and better mimic the topography of native ECM. The incorporation of collagen into the synthetic PET/PDMS scaffold improved cytocompatibility, as evidenced by increased HUVECs viability and spreading. These structural and compositional optimizations position the membrane as a functional surrogate for biological basement membranes in microfluidic environments. Although no direct quantification of solute permeability was conducted, the study evaluated the composite barrier’s performance using co-culture of endothelial (HUVECs) and glial (C6) cells under a continuous laminar flow. Fluorescence-based live/dead staining demonstrated high cell viability and successful cellular localization on opposite sides of the membrane after 24 h, indicating that the nanofiber interface supported bidirectional cell attachment without delamination or leakage. Additionally, numerical simulations performed using COMSOL Multiphysics highlighted the impact of membrane surface roughness on local shear stress distributions and fluid velocity profiles—parameters intrinsically linked to barrier stability and function in physiological systems. The combined biological and computational data substantiate the structural integrity and biocompatibility of the collagen-enriched nanofiber interface, supporting its potential use in future barrier modeling and transport studies.

Cherwin et al. [35] employed a dual flow organotypic microfluidic model to simulate intestinal barrier disruption via luminal exposure to bacterial collagenase over 48 h. The treatment induced dose-dependent degradation of collagen type I in the subepithelial ECM, accompanied by reductions in claudin-1 expression and aberrant goblet cell morphology, including diminished mucin production and altered cellular shape. Although no numeric permeability coefficient was provided, the observed diffusion of 10 kDa dextran and loss of tight junction markers indicate a substantial impairment of barrier function. These findings support the hypothesis that structural integrity of the collagen-rich ECM is critical to maintaining epithelial barrier stability in microfluidic intestinal models.

In a recent preprint, López García et al. [97] presented a collagen-based gut-on-chip model incorporating a native ECM interface. Although not yet peer-reviewed, this work proposes a quantitative methodology to assess the independent barrier function of a collagen type I membrane under microfluidic conditions, offering an alternative to synthetic membrane platforms. This fibrillar collagen interface supports epithelial monolayer differentiation and mimics the in vivo structural properties of the intestinal barrier. The authors performed quantitative fluorescein-based permeability assays under three conditions: (i) bare collagen membrane; (ii) collagen with a differentiated epithelial layer; (iii) infected epithelium. The findings demonstrated that the collagen membrane alone exhibits baseline permeability consistent with previously reported collagen-based systems. Importantly, the presence of a Caco-2 epithelial monolayer significantly decreased the permeability coefficient, thereby confirming the barrier function of the combined interface. Although prior gut-on-chip systems have used synthetic membranes, this work is among the first to explicitly isolate and quantify the independent contribution of a collagen type I matrix to barrier integrity under continuous perfusion in a microfluidic configuration. These results underscore the value of native ECM materials in advancing physio-mimetic barrier models for gut research.

The microfluidic-transwell hybrid system developed by Cenhrang et al. [69] demonstrated effective barrier functionality by integrating an electrospun collagen type I scaffold that supported Madin–Darby canine kidney (MDCK) cell monolayer formation under dynamic flow conditions. Barrier integrity was assessed using TEER measurements and tight junction gene expression (claudin-1, occludin), which increased under different flow rates (reaching 278 Ω·cm^2^, at 0.2 mL/min) and physiological shear stress. Additionally, selective transport was confirmed via apparent permeability assays using model compounds (caffeine and digoxin), showing values in line with in vivo and transfected cell line data. These findings validate the device’s capacity to maintain epithelial monolayer integrity while allowing regulated molecular passage, making it a relevant in vitro platform for transport and permeability studies.

The incorporation of collagen type I hydrogels as structural and biochemical components in microfluidic OoC systems presents significant implications for the assessment of barrier integrity. In this context, Ugodnikov et al. [98] evaluated the impact of collagen type I within a bilayer microfluidic platform designed for electrical cell–substrate impedance spectroscopy (MP-ECIS). When collagen hydrogel was introduced into the abluminal channel of the device, conventional TEER measurements using chopstick electrodes exhibited significantly elevated baseline resistance values, even in the absence of cells. Specifically, the addition of collagen hydrogel resulted in a TEER increase of up to 1723 Ω, yielding an apparent resistance of ~903 Ω·cm^2^, a level that exceeds the <100 Ω·cm^2^ usually reported TEER values for hCMEC/d3 brain endothelial cell line. These findings underscore the high intrinsic resistance of collagen matrices and their capacity to mask the contribution of the endothelial monolayer to barrier integrity.

### 5.2. Cellular Interface

A principal advantage of collagen type I as a barrier interface in microfluidic devices lies in its capacity to provide a native ECM substrate rich in integrin-binding sites, thereby facilitating cell adhesion, spreading, polarization, and, in some architectures, migration across or along the interface.

HUVECs and C6 glial cells were cultured on opposite surfaces of a dual-channel microfluidic platform developed by Eslam et al. [19], demonstrating robust adhesion and viability under continuous perfusion at 10 μL/min. Fluorescence microscopy confirmed confluent, well-adhered cell layers on both sides. Collagen integration significantly enhanced hydrophilicity, biocompatibility, and surface roughness, as evidenced by contact angle reduction and cytocompatibility assays. The combination of experimental data and computational simulations confirmed the interface’s stability under shear stress, supporting its suitability for applications in vascular and neural barrier models. This study represents a substantial advancement in the development of collagen-based nanofiber barrier membranes for dynamic cell culture systems.

In the CHIPS platform developed by Shiwarski et al. [58], 3D-bioprinted constructs of collagen type I were engineered with embedded, perfusable lumen channels containing vascular cells distributed within the ECM. These structures support active cell migration and ECM remodeling away from the lumen boundary into the surrounding collagen. The collagen-lumen interface operates as a dynamic scaffold facilitating capillary-like network formation and the polarization of vascular cells in response to hemodynamic and biochemical cues. Notably, the system exhibits size-dependent diffusion of soluble factors from the perfused lumens into the adjacent matrix, establishing molecular gradients that spatially guide directed cellular behaviors. As a whole, the interface functions as a tunable microenvironmental barrier that governs cell adhesion, migration, and polarization along structured paths. The platform further allows modulation of these interactions through lumen geometry and matrix stiffness, highlighting its relevance for modeling vascular development and tissue microenvironments.

Ghobadi et al. [15] developed a microfluidic platform integrating collagen type I hydrogels reinforced with BG NPs to support 3D cell culture. L929 fibroblasts were encapsulated within the collagen–BG NPs matrix and loaded into the central gel channel of the microfluidic chip. Viability assessments using live/dead staining demonstrated sustained cell survival over three days, with cells maintaining a rounded morphology consistent with biocompatible hydrogel encapsulation. Although the system was operated under static culture conditions, the device geometry with adjacent media channels facilitated nutrient diffusion, emulating aspects of perfused microenvironments. These findings suggest that the collagen–BG NPs scaffold supports cell viability and matrix compatibility within geometrically constrained microchannels, underscoring the adhesive competence of collagen interfaces in microfluidic applications.

Komsa-Penkova et al. [99] investigated the adhesion dynamics of adipose-derived mesenchymal stem cells (ADMSCs) on native and glycated collagen type I coatings under both static and dynamic (shear flow) conditions using a microfluidic BioFlux platform.

Their findings revealed rapid initial cell attachment within 3–5 min under flow across all substrates. Notably, while initial adhesion to glycated collagen—particularly collagen glycated for 5 days—was enhanced, subsequent exposure to increasing shear stress (i.e., 20 dyn/cm^2^) induced significantly higher cell detachment compared to native collagen (with adhesion values in the range of 27–37% for glycated collagen vs. 12% for native collagen). These effects were attributed to glycation-induced modifications in the collagen surface, including reduced elasticity, altered surface charge, and diminished adhesive forces, as confirmed by atomic force microscopy. Additionally, static adhesion assays showed early but transient increases in adhesion to glycated substrates, followed by reduced cell spreading and focal adhesion formation over time. These results underscore the importance of collagen modification state in regulating MSCs behavior at barrier interfaces and highlight critical design considerations for microfluidic systems employing collagen-based substrates in regenerative and diagnostic applications.

The study by González-Lana et al. [17] investigates the use of collagen type I as a structural matrix and surface-immobilized protein within COP-based microfluidic devices. To address challenges associated with hydrogel contraction by embedded contractile cells, the authors employed surface modifications including PAA-PG to enhance collagen immobilization. Their results demonstrate that collagen type I, when covalently bonded via PAA-PG treatment, supports long-term 3D culture of fibroblasts and glioblastoma cells by resisting hydrogel collapse. Confocal imaging confirmed homogeneous cell distribution and structural integrity for 13–20 days under continuous perfusion. Furthermore, the collagen interface enabled the generation of necrotic cores under high cell density, simulating ischemic conditions. These findings highlight the critical role of collagen type I as a functional interface capable of maintaining multicellular assemblies and localized cell–matrix interactions in perfused microfluidic environments.

### 5.3. Mechanical Separation and Structural Support

Beyond biochemical functionality and selective permeability, collagen type I barrier interfaces play a crucial role in the mechanical integrity and compartmentalization of microfluidic systems. Within biomimetic devices, these interfaces must sustain fluidic shear forces, resist transmembrane differential pressure, and maintain dimensional and spatial stability over time. Their ability to function as mechanical separators—preserving the physical distinction between apical and basal compartments or perfused and non-perfused zones—is essential for accurate simulation of tissue environments. Moreover, flow-induced deformation, delamination, or interface fatigue may compromise experimental reproducibility, cellular behavior, and molecular transport dynamics. Thus, collagen interfaces must combine biological compatibility with sufficient mechanical resilience to withstand continuous perfusion, directional shear gradients, and surface tension effects without structural failure.

While traditionally viewed as soft and compliant, recent advances in collagen processing and hybrid interface design have begun to address these limitations, enabling more stable and mechanically robust collagen-based barrier architectures in microfluidic platforms.

In a recent study, Eslam et al. [19] investigated a composite nanofibrous membrane composed of collagen, PET, and PDMS integrated into a dual-channel microfluidic chip. Through computational fluid dynamics simulations, they demonstrated that increased surface roughness—arising from collagen incorporation—resulted in nonuniform shear stress distributions, with elevated local shear rates near the interface. Furthermore, the flow rate was shown to significantly modulate both the velocity and shear profiles across the membrane. Experimental validation using human endothelial (HUVECs) and glial (C6) cell co-cultures confirmed high viability (~98%) on the opposing side of the interface after 24 h of shear exposure, indicating preserved structural integrity and functional separation. These results highlight the dual role of collagen-modified nanofiber membranes in modulating hydrodynamic shear while maintaining mechanical robustness, supporting their utility as biologically active, mechanically stable interfaces in microfluidic tissue models.

In a recent study, Shiwarski et al. [58] developed collagen type I scaffolds that are internally perfusable, enabling the formation of microfluidic structures with open, precisely defined lumens. These collagen constructs, termed CHIPSs, were fabricated with notable geometric accuracy, exhibiting less than 10% deviation from the original computer-aided design (CAD) models—an indication of their structural fidelity. When subjected to continuous perfusion with culture medium for over 24 h, the lumens remained patent and retained their architectural features, suggesting an ability to withstand moderate shear stress without undergoing deformation. This sustained lumen stability under flow conditions, along with their resistance to collapse, points to the mechanical integrity of the scaffolds and supports their use as passive architectural elements in engineered tissue systems. Moreover, successful co-culture of epithelial and endothelial cells under perfusion conditions confirmed not only the mechanical durability of CHIPSs but also their suitability for functional cellular integration over extended periods.

González-Lana et al. [17] provided in their study a clear demonstration that anchoring collagen type I to COP walls is not just a chemical refinement, but a mechanical necessity for preserving compartment geometry under cell-generated stress. In their COP devices, human cardiac fibroblasts embedded within collagen hydrogels rapidly deformed and collapsed the gel unless the barrier interface was robustly bonded. They showed that PAA-PG consistently outperformed APTES-based silanization and PDL immobilization treatments, maintaining >90% area for days at low collagen content (1.2 mg/mL) and extending stability to weeks at higher content. One should note that PAA-PG surfaces retained more collagen under high wall shear than the other chemistries, reinforcing the idea that strong interface coupling mitigates both tangential (flow) and internal (cell contractility) loads. In microfluidic terms, such mechanical separation is what keeps the hydrogel barrier from detaching or necking, preserves diffusion pathways, and sustains long-term co-culture scenarios in which fibroblasts would otherwise erase the intended architecture. Practically, this means that selecting PAA-PG (or an equivalently strong anchoring strategy) is integral to the structural support function of collagen type I interfaces: it maintains the designed geometry against cell traction, reduces collapse-induced cross-talk between compartments, and safeguards the fidelity of flow-defined gradients—even when the link to shear is indirect—by preventing matrix retreat from the channel walls.

### 5.4. Biochemical Modulation

The premise is simple but powerful: when collagen type I is cast as the barrier interface in a chip, it does more than “hold cells in place.” It binds ligands, shapes diffusive and advective gradients, and clusters integrins to rewire signaling—often on timescales faster than we tend to measure. In practice, this means that the same microchannel geometry that delivers a chemokine gradient also sets the local collagen fiber orientation and pore size, which in turn biases receptor engagement, matrix sequestration of cues, and ultimately barrier selectivity.

**Figure 12:**
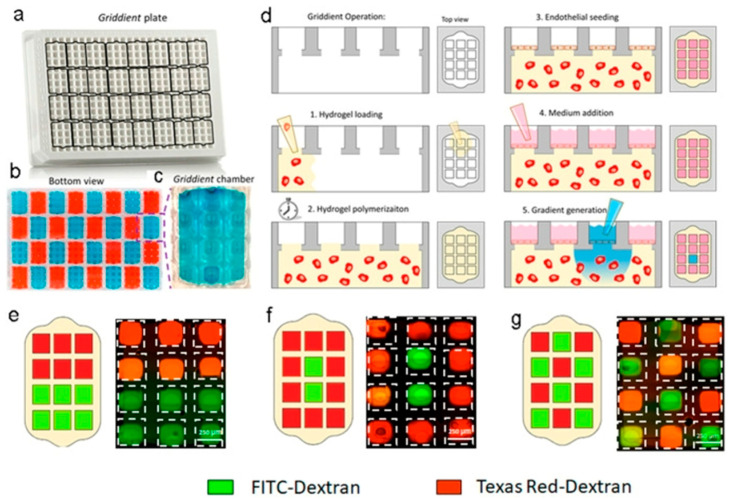
Griddient operation: (**a**) Image of the Griddient platform, black outlines show a 32 individual microfluidic chambers underneath the platform. (**b**,**c**) Picture of the bottom of the Griddient platform showing the microfluidic chambers filled with food dye for visualization purposes. (**d**) Experimental protocol for the Griddient operation: (1) Cells were mixed with a type I rat tail collagen solution and injected into the main chamber through one of the diffusion ports; (2) Collagen polymerized for 10–15 min at RT; (3) When the collagen is fully polymerized, endothelial cells are seeded on top of the hydrogel interface as a monolayer; (4) After endothelial cells have been attached, media or PBS, were added to the reservoir wells; (5) The media/PBS of each well can be modified to generate or revert the desired gradients; (**e**–**g**) 40 kDa FITC-Dextran and 70 kDa TR-dextran diffusion in the Griddient after 24 h in lineal (**e**), radial (**f**), or periodic (**g**) configuration. Reproduced with permission from [100].

The “griddient” microfluidic device proposed by Sanches-de-Diego et al. [100] exemplifies how a collagen type I barrier can behave as an active biochemical modulator rather than a passive container. The device organizes 32 gel-filled chambers, each coupled to a reservoir by a bottom diffusion port that doubles as a capillary valve, enabling manual loading and stable, reconfigurable gradients across a 3D collagen matrix seeded with cells (Figure 12).

In practice, cells are mixed with rat-tail collagen type I, polymerized in situ within minutes, and then exposed to nutrient or drug differentials imposed from the reservoirs; gradients stabilize within hours and remain steady over days while the gel maintains its integrity. Importantly, the collagen type I interface serves as a transport resistor—and, effectively, a biochemical sponge—because the gradient shape depends on hydrogel architecture and the gel-to-reservoir ratio, and can be further biased by reservoir volumes that induce convective components. The reported results underscore that the collagen type I barrier is integral to gradient formation and signal presentation, tuning how molecules are delivered, retained, and sensed by embedded tumor cells—precisely the biochemical modulation role demanded in microfluidic studies.

Using a “piggyback-style” microfluidic setup in which channel geometry intrinsically aligns collagen type I fibers, Lichtenberg et al. [90] placed an external morphogen source across the tissue chamber to build a stable gradient, enabling simultaneous but orthogonal biochemical and structural cues.

In this context, luminal epithelial cysts displayed directional ruffling and protrusions only when two conditions were met: a sufficient hepatocyte growth factor (HGF) gradient (both mean and slope mattered) and an aligned collagen interface. Under these combined cues, protrusions and subsequent elongation tracked the fiber axis; when fibers were random—or when gradients were sub-threshold—protrusions scattered, elongation diminished, and branches effectively stalled. Mechanistically, the response depended on cadherin-3: CDH3-null cysts failed to polarize or elongate despite exposure to the same gradients and matrices, indicating that the collagen type I barrier interface modulates chemokine guidance by organizing adhesion and downstream mechanotransduction at the interface. In short, the biochemical gradient “works as intended” only when the collagen architecture presents it in a guidance-competent form—underscoring that soluble cues and ECM structure are inseparable determinants of epithelial pathfinding.

**Figure 13:**
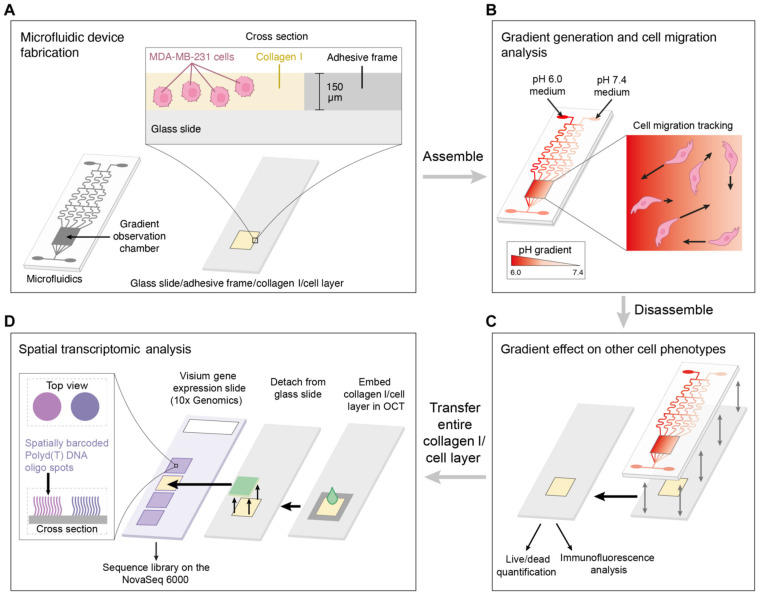
Integrated microfluidic workflow: (**A**) The microfluidic device was cast in PDMS on a clean room microfabricated mold. The cells were cultured in a 150-μm-thick collagen I layer deposited on a glass slide to match the gradient area; (**B**) The PDMS microfluidic device and the glass slide containing the collagen I/cell layer were assembled in a metal frame holder and perfused with growth media of pH 7.4 and 6.0 through the inlet channels, in a CO_2_ incubator or in the environmentally controlled chamber of an epifluorescence microscope. This allowed rapid formation of a constant pH gradient across the observation chamber, through which cells could be monitored by live imaging and migration parameters analyzed; (**C**) Disassembly of the device could be performed without damage to the cells in the collagen I layer, which were subsequently analyzed using either live/dead staining or IF analysis. (**D**) Alternatively, the collagen I/cell layer was frozen in OCT compound and transferred as an OCT/collagen I block to a GEX slide for spatial transcriptomic analysis. Reproduced with permission from [101].

Auxillos and colleagues [101] engineered a resealable microfluidic platform that lays a steep pH gradient (≈7.4 → 6.0 across 5 mm) directly over cancer cells immobilized within a 150-μm collagen type I layer, enabling continuous live imaging and, after disassembly, spatial transcriptomics on the same specimen (Figure 13).

The collagen interface is not a bystander: by pinning cells in a defined diffusion path and mediating proton transport through the gel, it shapes the local acid–base landscape experienced by each cell, bringing the in vitro field closer to tumor-like micro gradients. Within hours, cells exhibit phenotype shifts that map onto pH—reduced short-term motility toward acidic regions and a decline in apoptosis markers—and, crucially, spatial gene-expression programs that vary monotonically with position along the gradient. In effect, the collagen type I barrier translates a physical-chemical cue (H^+^) into stratified signaling states over hundreds of microns. Because the gradient is rapidly tunable and the device is reversible, the same collagen-defined interface supports both dynamic modulation and high-content readouts, underscoring how ECM architecture and diffusional transport co-govern biochemical guidance in microfluidic models.

**Figure 14:**
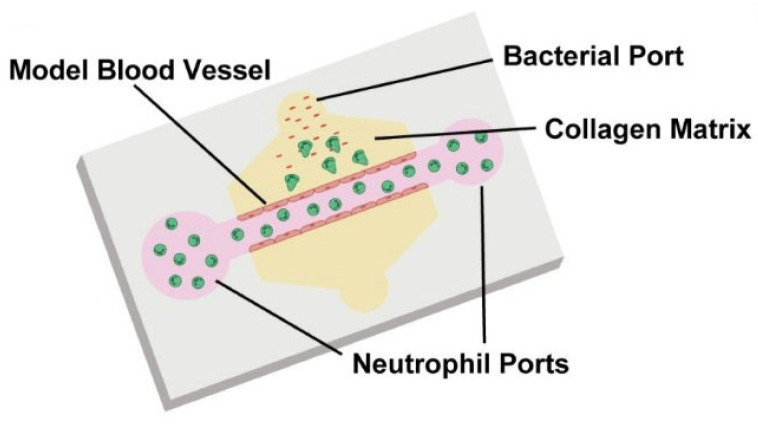
Schematic of the microfluidic system used to model tissue infection near a blood vessel with varied collagen concentrations. Reproduced in part with permission from [102].

In an infection-on-a-chip model [102], endothelialized lumens were cast in collagen type I across a (2–6 mg/mL) range (Figure 14). Primary human neutrophils circulating within the vessel were stimulated by Pseudomonas aeruginosa introduced into the surrounding matrix.

Under these conditions, collagen concentration alone governed the immune response: extravasation peaked in medium-density gels (4 mg/mL), whereas post-transendothelial migration was fastest and farthest in the most open networks (2 mg/mL), declining progressively as collagen increased. Notably, vessel formation and barrier integrity were similar across the series, with comparable endothelial cell density and no collagen-dependent leakage, indicating that the effects were not simply due to permeability differences. Consistent with this, endothelial cytokine secretion profiles did not track the extravasation trend, arguing against a purely soluble-factor explanation and pointing instead to matrix-mediated modulation. In this configuration, bacterial stimuli established chemotactic fields in the gel, while the collagen network set pore geometry and integrin engagement at the barrier.

**Figure 15:**
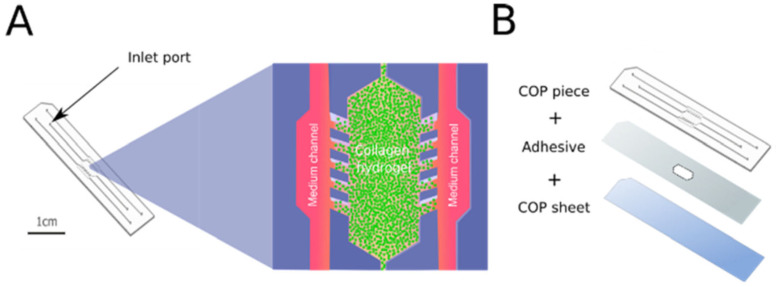
Microfluidic device appearance and components: (**A**) Magnified representation of a COP-MD with collagen hydrogel embedded cells (green) confined into the central chamber and culture medium perfused through the two lateral channels creating a gradient of medium; (**B**) Schematic illustration of the elements integrating the microfluidic device. Reproduced with permission from [17].

Highly contractile cells can quickly remodel or even collapse collagen hydrogels in microfluidic chambers, erasing compartmental boundaries and covering intended chemical gradients. González-Lana and colleagues [17] tackled this by chemically securing the collagen–chip interface in COP devices (Figure 15).

Using PAA-PG, they created covalent linkages that firmly anchored collagen type I to COP walls, which preserved 3D geometry during long-term culture of fibroblasts. In practice, PAA-PG retained >90% of the hydrogel cross-section for days to weeks (depending on collagen concentration), stabilizing the barrier that shapes biochemical signaling fields. The study thus underscores a practical but often overlooked principle stating that biochemical modulation by collagen barriers is only interpretable when the barrier is mechanically and chemically secured.

The study of Nakayama-Kitamura et al. [103] is another example of extracellular-matrix signaling. Adding collagen type I microfibers (CMFs) to the gel was shown to accelerate capillary network formation and maturation by supporting astrocyte survival, and the effect depended on β1-integrin engagement. The collagen interface did more than just “host” the neurovascular unit. Its microscale fibrillar architecture engaged integrins on astrocytes, and tuning collagen’s microstructure reshaped the biochemical conversation among barrier constituents.

## 6. Applications

### 6.1. Organ-on-Chip Models

OoC platforms have matured into modular, automatable testbeds for building disease models and screening candidate therapeutics directly in human-relevant microenvironments. By deliberately juxtaposing tissue–tissue and even organ–organ interfaces, these microphysiological systems capture key features of human organ microenvironments, such as 3D architecture, dynamic cell–matrix exchange, and relevant biophysical cues—shear, stretch, and cyclic strain—that continuously write and rewrite cell state. In comparison with conventional 2D assays, chips typically deliver higher physiological fidelity while also softening the usual pain points of preclinical research, from species gaps and long timelines to expense and ethical constraints.

Within this landscape, collagen type I serves as more than a passive scaffold. Cast as a barrier interface, collagen type I shapes the porosity and ligand presentation that govern transmural transport, tunes integrin-dependent signaling that underlies barrier maturation, and constrains gradient formation by resisting cellular contraction and flow-induced remodeling. Whether deployed in its native fibrillar form, blended with synthetic polymers to stabilize mechanics, or micro-engineered into defined fiber architectures, collagen type I becomes a handle for modulating both biochemistry (chemokine diffusion, matrix-bound cues) and mechanics (stiffness, pore geometry) under flow. The recent literature (2023–2025) increasingly treats this interface as a controllable variable—one that can accelerate microvascular assembly, gate immune cell trafficking without altering endothelial leak, or maintain long-lived, self-organized nutrient and oxygen gradients. Framed this way, the most informative chip studies are not merely “using collagen”; they are engineering a collagen type I barrier to interrogate how matrix properties translate into transport phenotypes and cell–cell communication across human microtissues.

#### 6.1.1. Gut-on-Chip

**Figure 16:**
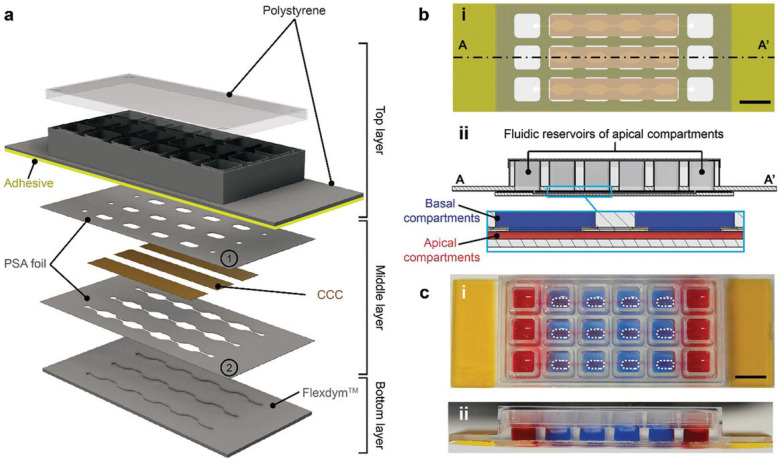
(**a**) Schematic representation of the individual layers of the MultiU-Int microfluidic chip, their arrangement, and materials. The top layer was a commercially available, multi-well slide made from polystyrene plastic material. The middle layer of the chip was fabricated by sandwiching CCC strips between two PSA foils. The upper foil (①) featured oval areas where fibroblasts and, later, immune cells were allowed to interact with the IEB model (fibroblast seeding areas). The lower foil (②) featured the same pattern of channel structures that were hot-embossed into the bottom layer of the chip. The bottom layer was fabricated by hot embossing the elastomer Flexdym. (**b**) Schematic cross-sections of the MultiU-Int microfluidic chip showing details of the layer alignment: (i) bottom view, and (ii) side view; ((**c**)-(i)) Top view, and ((**c**)-(ii)) side view photographs of the MultiU-Int microfluidic chip. For visualization, apical channels and their reservoirs were filled with red fluid, and the basal compartments were filled with blue fluid. The cell seeding areas on the basal side are marked with white dashed lines. Scale bars: 10 mm. Reproduced with permission from [104].

Nguyen and colleagues [104] built a multi-unit intestine-on-chip system in thermoplastics (to avoid compound absorption and medium evaporation) that replaces stiff polymer films with an elastic collagen type I membrane formed from native, non-crosslinked collagen fibers (Figure 16).

The epithelial layer reached tight, polarized barriers within a week and could be challenged with immune cells and inflammatory cues in defined compartments. The collagen membrane supported rapid barrier maturation and on-chip remodeling, enabling stepwise inflammatory bowel disease-like perturbations without the confounding mechanics of PET/PC inserts. The platform is directly usable for studies that need immune–epithelium crosstalk on a bioactive basement-membrane analogue, and it highlights manufacturable routes (thermoplastics + collagen) that sidestep PDMS sorption. As indicated by the authors, experiments with the system are currently limited by low temporal resolution. This constraint could be substantially alleviated by either (i) integrating online sensors—e.g., for transepithelial electrical resistance, oxygen, and cytokine concentrations—directly into the microfluidic chip, or (ii) implementing an automated tilting-and-imaging setup. With these upgrades, the system would support detailed analyses of cell–cell interaction dynamics, the emergence of pathogenic morphologies, and inflammatory signaling.

López Garcia et al. [97] reported in their preprint on the fabrication of thin, porous collagen type I membranes and integrated them as the central barrier in a gut-on-chip. They characterized thickness and transport, and used the platform to probe epithelial barrier function and host–pathogen interactions. This work is a concrete blueprint for sub-100 µm collagen membranes that couple optical access with realistic permeability—well suited for drug transport and infection models that benefit from ECM-derived ligand display. Clinically aligned adoption will hinge on lot-to-lot consistency, sterilization that preserves bioactivity, and durability under long-term flow.

**Figure 17:**
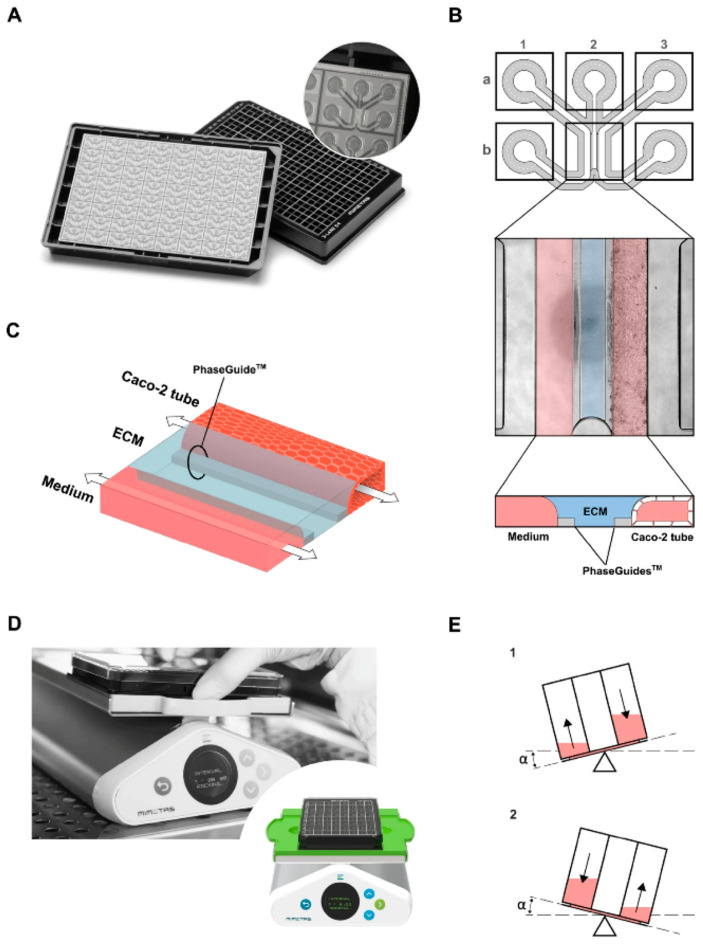
(**A**) Photograph of the bottom and top of the OrganoPlate, showing 64 microfluidic channel chips embedded in a standard 384-well microtiter plate and a zoomed-in view of a single chip. (**B**) Schematic picture depicting the structure of a single microfluidic chip, which consists of three channels: the left channel (1), middle channel (2) and right channel (3), which are accessible via inlets (1a, 2a, 3a) and outlets (1b, 3b). The right channel is where the Caco-2 cells form the epithelial tubule, the middle channel is seeded with ECM, and the left channel is filled with medium. The right and left channels are separated from the middle channel by PhaseGuides, which allow barrier-free channel separation. (**C**) 3D structure of a perfused chip. (**D**) Photographs of the OrganoFlow device, on top of which OrganoPlates are placed to induce medium flow. (**E**) Transversal view of a channel that shows how bidirectional medium flow is generated by continuous angular tilting of the OrganoPlate by the OrganoFlow rocker. Reproduced with permission from [91].

Morelli et al. [91] used the membrane-free OrganoPlate to form Caco-2 tubules seeded against collagen type I ECM, then quantified toxin-specific damage with TEER, imaging, and cytotoxicity assays (Figure 17).

After exposure of the model to toxins, the setup demonstrated dose-dependent reductions in barrier permeability, delivering higher sensitivity than conventional static models and ran in a 64-chip format suitable for screening. It is important to note that the epithelial barrier developed directly against collagen type I, giving diffusion and adhesion conditions closer to subepithelial matrix. The work is immediately translatable to toxicology and food safety screens and shows that collagen-based microenvironments can boost assay sensitivity while remaining compatible with automation and multi-parametric readouts.

#### 6.1.2. Blood–Brain Barrier

Effective treatment of brain tumors is hindered by intrinsic drug resistance and the limited permeability of the BBB.

In short, the chip illustrates how a collagen-based BBB interface does not merely “sit between” compartments; it conditions the biochemical conversation—cytokines, EMT signaling, angiogenic cues—through which glioblastoma multiforme microenvironment and the neurovascular unit co-evolve, and it provides practical levers (controlled opening, defined spacing) to probe that dialogue with mechanistic precision.

**Figure 18:**
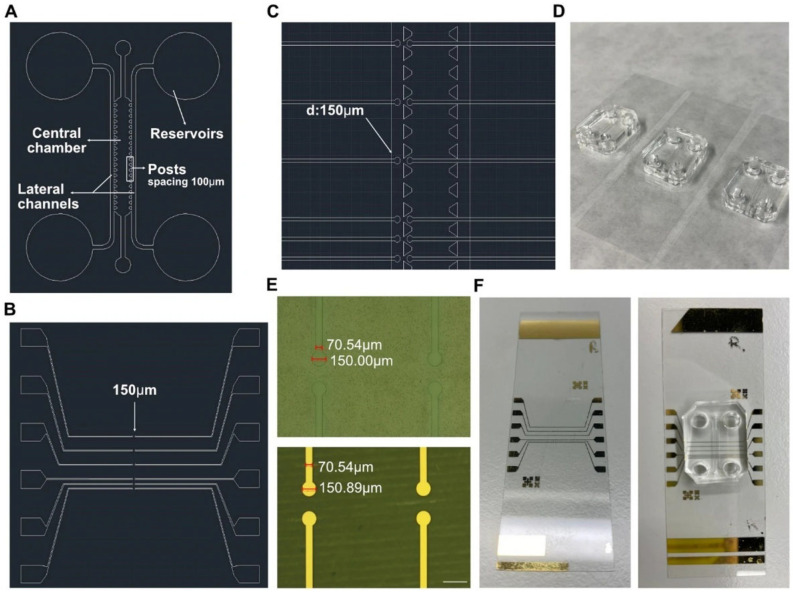
BBB on chip and measuring system design and fabrication: (**A**–**C**) AutoCAD (AUTODESK, San Francisco, CA, USA) picture of the microfluidic design, electrodes design and its arrangement with the BBB-oC, respectively. (**D**) Fabricated BBB devices suitable for cell seeding. (**E**) Optical images before and after Au/Cr evaporation on the lithographed design. Scale bar 250 µm. (**F**) TEER-BBB ready for cell incorporation and resistance measuring. Reproduced with permission from [44].

Palma-Florez and colleagues [44] integrated micrometric TEER electrodes into a tri-culture BBB chip (human endothelial cells co-cultured with pericytes and astrocytes) and used a 3D ECM setup in which the endothelial channel was collagen type I–coated and the central hydrogel compartment hosted pericytes (Figure 18). It was observed that from day 4 to day 10, TEER rose ~2.3-fold, from 5400 to 12,480 Ω·cm^2^.

The platform delivered impedance readouts during barrier maturation and was then used to quantify the permeability and endothelial effects of multi-functionalized gold nanorods (GNRs) targeting Alzheimer’s disease. After injection of polyethylene glycol (PEG)-functionalized GNRs co-conjugated with angiopep-2 peptide and the D1 peptide (β-amyloid fibrillation inhibitor), the Nyquist curve shifted to higher Z′ (12,480 → 14,352 Ω·cm^2^, day 7 to day 8), indicating elevated TEER; this suggested reinforcement of endothelial tight junctions and a concomitant decrease in BBB permeability. The practical takeaway is straightforward: collagen type I at the blood-facing interface provides a stable, bioactive surface for endothelialization, while the on-chip TEER geometry gives interpretable, location-aware electrical measurements—a combination that is immediately usable for screening nanoparticle transport and monitoring barrier integrity over time.

**Figure 19:**
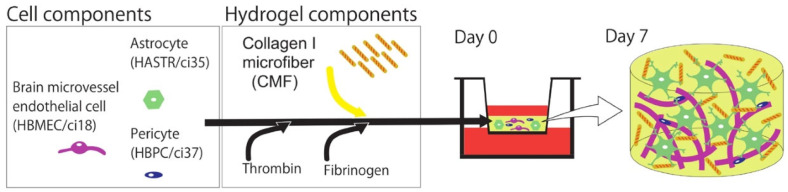
CMFs are important for the autonomous production of capillary networks: Schematic of the BBB–NET components. Reproduced in part with permission from [103].

Nakayama-Kitamura et al. [103] advanced a 3D BBB microphysiological system by incorporating collagen type I microfibers within the hydrogel to guide capillary network formation (Figure 19).

The approach improved endothelial organization and supported networked BBB-like structures under perfusion. In application terms, this moves beyond a flat monolayer: collagen type I is not merely a coating but an architectural cue that shapes microvascular topology and, by extension, diffusion paths, and shear exposure. The implication for next-generation BBB chips is that tuning collagen fiber content and alignment can be used intentionally to steer angiogenesis and junctional maturation—valuable knobs for disease modeling (e.g., inflammation-driven leak) and for calibrating transport assays to physiologic ranges.

**Figure 20:**
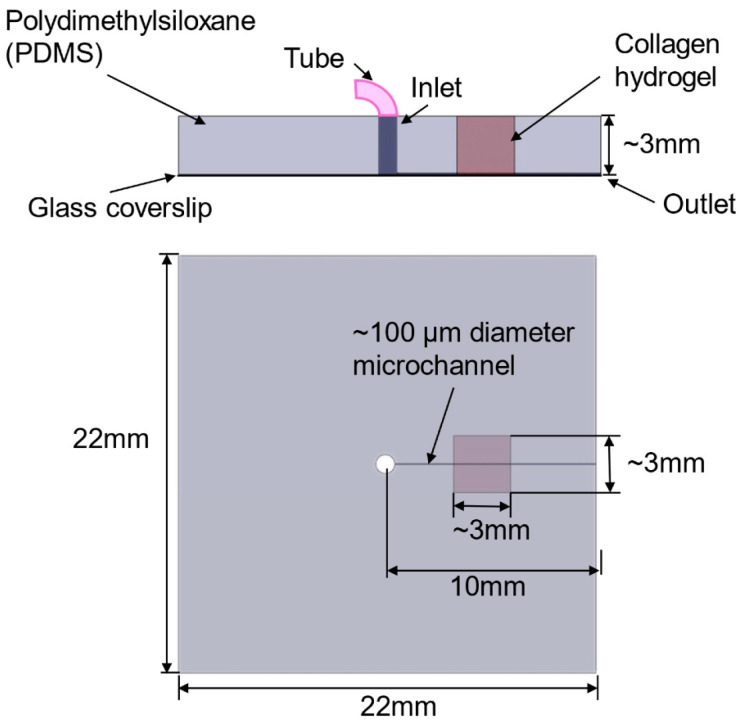
Schematic of the PDMS–collagen hydrogel microchannel device. PDMS solidification on a glass coverslip to form a 22 mm × 22 mm × ~3 mm base. A microchannel with a diameter of ~100 μm was formed by pulling out a microneedle from the collagen hydrogel. The inlet was formed with an 18-gauge tubing adapter and connected to PE-50 tubing for perfusion, which was driven by a syringe pump. Reproduced with permission from [105].

Li and co-workers [105] built a circular microchannel BBB in a PDMS–collagen type I hydrogel (≈4–5 mg/mL; genipin-stabilized), seeded with human cerebral microvascular endothelial cells and perfused at venule-like shear (Figure 20).

The device matched in vivo-like permeability to small and large solutes, and the team used it to probe how glycocalyx-modulating agents and inflammatory cues shift barrier function; they also quantified tumor-cell adhesion under flow. From an engineering perspective, this is a pragmatic blueprint for capillary-scale BBB models that require no cleanroom: the collagen type I wall functions simultaneously as the barrier interface and tissue compartment, supports physiologic hemodynamics, and permits high-resolution optical imaging. For future work, the same design can host astrocyte/pericyte layers on the collagen lumen or be extended to networks for compound screening.

#### 6.1.3. Skin-on-Chip

Researchers [106] have attempted to fabricate innervated skin tissues using collagen sponges, cell-culture inserts, and microfluidic devices to partially recapitulate the skin’s layered architecture. Nevertheless, a fully innervated, full-thickness skin model has not yet been realized. To mimic the in vivo neurite-penetration pathway, dorsal root ganglion neural spheroids were positioned at the base of the dermal layer, and neurite outgrowth was evaluated across multiple hydrogel formulations.

Miura and colleagues [106] engineered a 3D skin construct using a culture device with anchoring structures. It was found that the used collagen–Matrigel composite hydrogel markedly limited culture-induced tissue contraction, which often leads to detachment of the skin construct from the anchors during the culture.

In a complementary study, Ahn and colleagues [107] built a cleverly compartmentalized chip to interrogate epidermal–neuronal crosstalk. The device housed an acellular collagen type I hydrogel as the engineered “dermal” interface separating channels seeded with human keratinocytes and dorsal root ganglia neurons.

By tuning collagen concentration (1.5–3 mg/mL) and laminin content, they guided axonal extension through the collagen barrier toward a stratifying, air–liquid-interface epidermis, then quantified barrier integrity with fluorescein isothiocyanate (FITC)-dextran transport. Practically, this model gives researchers a way to test how an epidermal barrier that is physically supported by collagen type I shapes neurite growth and sensory function without the confounding remodeling that fibroblasts would introduce. For the barrier interface domain, the model is valuable because collagen density and composition at the interface jointly determine both permeability and axon-guidance cues. Moving forward, two immediate experiments appear tractable: (i) systemically varying collagen type I fibril architecture (e.g., shear-aligned versus isotropic) to test effects on neurite trajectory and leakage; (ii) replacing the single-material collagen type I barrier with a biphasic collagen/laminin veneer to better emulate the dermal–epidermal junction and interrogate selective transport.

Rhee et al. [108] advanced the technology toward pharmacology by creating a perfused, full-thickness skin-on-chip in which the dermis is a collagen type I hydrogel populated with fibroblasts, overlaid by a keratinocyte epidermis at ALI.

The micro-stereolithography-3D printed chip avoids PDMS absorption, and continuous perfusion through microchannels abutting the collagen dermis improved viability and stabilized cytokine release. Importantly, when the tumor necrosis factor alpha (TNF-α) and treated with dexamethasone, the chip’s IL-6/IL-8 pro-inflammatory cytokines resembled human skin biopsies more than 2D monocultures. From an application standpoint, the collagen type I dermis here is not only a structural compartment; it is the dose-routing medium that dictates how perfused small molecules spread, bind, and clear. This makes collagen formulation a first-order design parameter in drug testing: collagen concentration and cross-linking modulate diffusion–binding kinetics and, consequently, the apparent potency. To strengthen translational relevance, future iterations should (a) report quantitative permeability and partition coefficients for benchmark compounds across the collagen type I interface, and (b) include fibroblast-driven matrix remodeling readouts (e.g., matrix metalloproteinase activity, second-harmonic imaging of fibrils) so that drug responses can be interpreted in the context of evolving barrier microstructure.

Barros et al. [109] pushed toward intervention testing—microneedling-driven delivery and skin-cancer therapy—using a multilayered human skin/skin-cancer-on-chip platform. The fabricated skin model included vascular, dermal, and epidermal compartments; maturation markers in the dermis included collagen type I and fibronectin, while the epidermis exhibited filaggrin and keratin 10/14/19 stratification at ALI. In practice, the collagen type I-rich dermis serves two roles: it resists microneedle penetration and then guides solute transport through the wound tract; in the cancer variant, it also modulates tumor–stroma mechanics that govern intradermal drug spread. This is precisely where the platform aims for application-oriented studies—researchers can vary needle geometry, quantify insertion force, and map payload distribution against a reproducible collagen interface under flow. What would truly accelerate progress are standardized, post-microneedling barrier recovery metrics—TEER, tracer permeability, and fibril re-assembly kinetics—paired with head-to-head comparisons of collagen formulations (native, remodeled, and composite chitosan–collagen) to define how dermal matrix state governs both efficacy and safety of percutaneous delivery.

#### 6.1.4. Liver-on-Chip

**Figure 21:**
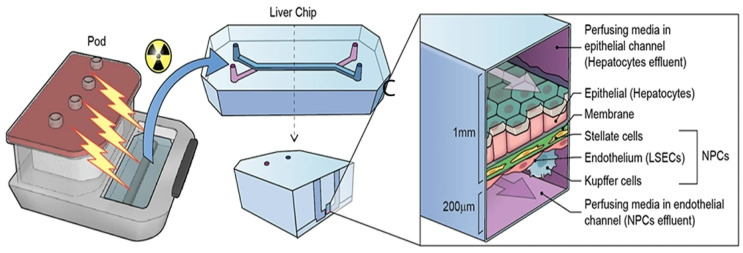
The Emulate Liver quad-culture chip in which hepatocytes are grown on the upper channel while LSECs, HSCs and Kupffer cells are grown on the lower channel. These channels are separated by a semi-permeable membrane which allows communication among the groups but does not allow cell migration from one channel to the other. Each channel receives separate perfusing media at a defined and controllable flow rate which replicates shear stress in normal liver. Reproduced in part with permission from [110].

Ueda and colleagues [110] used the commercial Emulate platform to construct a liver quad-culture chip (primary human hepatocytes over a separate endothelial channel containing liver sinusoidal endothelial cells (LSECs), Kupffer, and hepatic stellate cells)) and set out to determine whether the model reproduces radiation-induced liver injury (RILD) and whether the antioxidant N-acetylcysteine amide can attenuate that response (Figure 21).

Mechanically, the “barrier” here is the porous membrane separating the two channels; functionally, its surface is rendered biological by a collagen type I/fibronectin coating before cell seeding. Importantly, the coating step proved critical: it stabilized adhesion on both sides of the membrane, sustained perfusion for up to a week, and yielded clear, cell-type–specific injury readouts—such as liver sinusoidal endothelial cell death and dysfunction, hepatic stellate cell activation, and hepatocyte metabolic shifts—that were largely muted in simpler co-cultures. In other words, when the membrane approximates a collagen-presenting basement-membrane interface, the model reveals multicellular injury phenotypes—and their therapeutic mitigation—that align with the biology of radiation induced liver disease.

A more engineering-driven study was reported by Baddal et al. [62]. They designed and characterized a two-channel liver chip fabricated with accessible methods (3D stereolithography printing + PDMS molding). Here, both channels were chemically activated and then pre-coated with collagen type I (~1 mg/mL) prior to seeding HepG2 hepatocellular carcinoma cell line in the upper “parenchymal” channel; the lower channel was reserved for endothelialization. The device ran under continuous flow and produced textbook outputs—albumin, alanine transaminase/aspartate aminotransferase, alpha-fetoprotein—at physiologically reasonable levels with low lactate dehydrogenase release, essentially validating that a collagen type I interface is sufficient to establish a stable hepatic monolayer in a low-cost prototype. The results underscore a practical point: for laboratories prototyping new chip layouts or flow regimens, collagen type I remains a reliable first-line ECM, supporting hepatocyte adhesion, survival, and liver-marker production under shear.

Ferarri et al. [111] designed, fabricated, and validated two liver-on-chip models to recapitulate key features of the hepatic sinusoid in vitro. The first, a direct-contact platform, permits immediate endothelial–hepatocyte communication. The second, an ECM-mediated-contact platform, separates the endothelial channel from a 3D hepatic construct with a ~100 µm collagen–fibrin layer, more closely approximating the in vivo space of Disse. Although developed in a vascularized-liver context, the devices are versatile: the same architecture can support organ models that require low seeding volumes and precise vascular modeling, including BBB and tumor microenvironment systems [98]. This methodology also opens avenues for coupling microfluidics with vascularized tissue—such as perfusing immune components through the vascular channel—and for integrating these units into multi-organ-on-chip platforms to probe crosstalk among vascularized organs.

Li et al. [112] introduced a microneedle-array-based strategy to build hepatic sinusoids within a liver-acinus-on-a-chip microsystem. Using a custom 3D-printed microneedle array as a mold, they first generated larger “primary” sinusoids for demolding. Subsequent microflow-guided self-assembly then yielded smaller “secondary” sinusoids. The resulting sequence—primary structures forming first, followed by gradual emergence of secondary networks—demonstrates a controllable route to reconstruct sinusoidal architecture in vitro.

In a gut–liver chip [113], the barrier/interface was deliberately asymmetric—fibronectin (1.6 µg/cm^2^) on the intestinal channel and collagen type I (1.6 µg/cm^2^) on the hepatic channel –which was enough to form tight intestinal monolayers. Under dynamic conditions, the platform tracked apical-to-basolateral transfer of parent drugs alongside time-resolved appearance of triazolam, diclofenac, bufuralol, and propranolol, with metabolite levels generally higher than in matched static cultures. In practical terms, this shows that a simple collagen type I coating on the hepatic channel can support end-to-end absorption–metabolism and interaction assays without primary hepatocytes, provided sampling is done on both channels. Two cautions temper immediate translational use: bidirectional rocking is not physiological perfusion, and PDMS sorption substantially confounds lipophilic substrates, which can depress parent levels and scramble quantitative metabolism. A natural extension within the same architecture would be to keep the intestinal side as is and treat the hepatic interface as an experimental variable—comparing the current collagen type I coating with thin composite matrices—to test whether barrier composition measurably sharpens hepatocyte function and metabolite formation under true perfusion, while preserving the tight neighboring epithelium.

#### 6.1.5. Lung-on-Chip

Gao and colleagues [85] built a microfluidic airway-on-chip whose wall is a compliant tube cast from collagen type I and lined with human airway epithelium under continuous warm, humid air perfusion (two weeks at ALI). Because the barrier is itself collagen, the wall deforms and wets like a native small airway. When the team drove clinically relevant ventilation waveforms through the lumen, repetitive airway collapse–reopening produced sharper epithelial injury and barrier leakage than simple overdistension. That very practical readout—injury mode as a function of ventilation strategy—immediately positions the device for head-to-head testing of ventilator settings and adjuncts (e.g., expiratory flow resistance) before animal studies. Methodologically, the work also shows that collagen-only tubes can be cultured stably at ALI and interrogated under sustained airflow, which has been a recurring challenge for flat-membrane systems. Future studies coupling this platform to primary patient epithelium, mucus rheology measurements, or aerosolized drugs would be straightforward and clinically relevant.

Park et al. [114] engineered an airway-on-chip with an ultra-thin matrix-derived membrane and imposed bidirectional airflow to mimic breathing cycles, rather than the usual unidirectional sweep. Under these conditions the airway epithelium developed a visible heparan sulfate-rich glycocalyx, along with more representative mucociliary differentiation, and showed flow-dependent differences in tight-junction and mucus markers. From an application standpoint, the key contribution is not the airflow per se, but that a collagen-rich biological interface supports formation of structures (glycocalyx) that are frequently absent on rigid synthetics. Consequently, these results unlock questions about infection, pollutant deposition, and shear-mediated drug responses that depend on the glycocalyx as a functional barrier. The immediate next step is to quantify how glycocalyx thickness and composition vary with collagen content/architecture in the membrane and whether those changes alter pathogen binding or nanoparticle penetration.

Licciardello et al. [81] miniaturized the “thick” portion of the alveolar barrier by sandwiching a fibroblast-embedded collagen type I hydrogel between an apical epithelial compartment at ALI and a basolateral endothelial channel. Although a polycaprolactone/gelatin electrospun sheet supplied structural support, the interfacial matrix experienced by the cells was collagen type I. The device maintained epithelial and endothelial tightness for ~10 days and responded to lipopolysaccharide with the expected epithelial junctional disruption—i.e., a functional inflammatory readout built on a collagenous interstitium. From a practical standpoint, this work addresses a persistent gap: whereas many lung chips capture only the “thin” gas-exchange barrier, incorporating a collagen type I interstitium permitted fibroblast–epithelium–endothelium crosstalk, which is the very substrate for tissue remodeling, edema formation, and drug transport.

Although not a microfluidic study per se, the organoid biobank reported by Ebisudani et al. [115] offers a practical blueprint for what a lung-on-a-chip built around a collagen type I barrier should actually model and measure: niche-factor dependencies that track with lineage state and therapeutic response. Looking ahead, collagen type I lung chips should incorporate lineage-switch controls to probe how matrix mechanics and soluble factors co-determine signaling molecules (i.e., Wnt-3A/R-spondin) status. Then, they should use those stratified platforms to evaluate combination regimens mirroring the therapeutic bifurcation proposed by the organoid study, grounding this in a barrier-interface model that captures transport, mechanics, and drug exposure under physiologically relevant flow.

Saygili et al. [116] developed a biomimetic microfluidic model integrated with an optical pH-sensor to simulate drug-induced lung injury and monitor cytotoxicity-related acidification of the culture medium. The interstitial region was recreated using a bacterial–cellulose-based vascular membrane positioned beneath a connective tissue layer emulated with gelatin methacrylate hydrogel. Reduced collagen type I secretion after nintedanib administration further supported the model’s relevance for in vitro disease modeling. To provide a holistic readout, fibrotic–progression-depended cytotoxicity was quantified in real time by the optical pH sensor, which could resolve subtle pH increases or decreases within a relatively large reservoir under dynamic flow.

#### 6.1.6. Oral Mucosa-on-a-Chip

**Figure 22:**
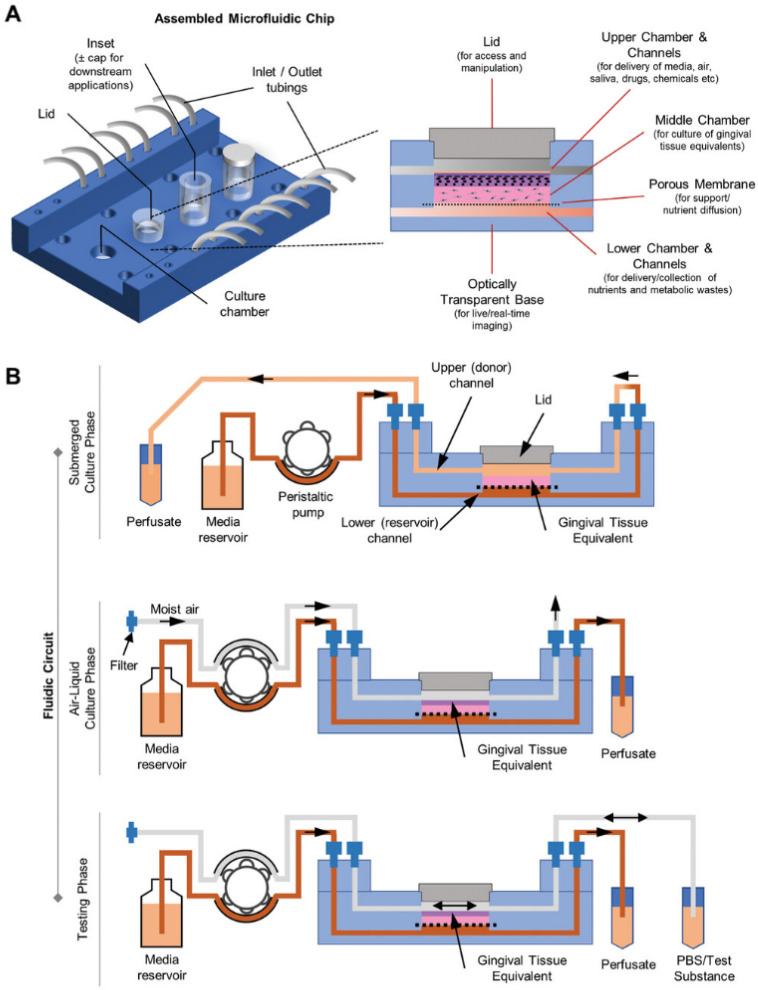
Schematic representation of microfluidic gingiva-on-chip device: (**A**) Schematic view of the assembled device that shows the different features such as microchannels, chambers, inlets, and outlets micromilled into PMMA sheets that are vertically stacked and thermally bonded; (**B**) Schematic representation of fluidic circuit used for perfusion of media, air, and test substances at different phases of the organotypic culture. Reproduced with permission from [117].

Muniraj and colleagues’ [117] vertically stacked gingiva-on-chip shows why flow and barrier context matter for practical readouts: air–liquid interface culture under perfusion yielded thicker, better-differentiated epithelium with intact basement membrane markers and measurable drug permeation (Figure 22).

The same device could be subjected to an injury-mimicking insult to model ulceration and then used to rank oral-care formulations by irritation potential—capabilities that are difficult to reproduce in static inserts.

Kim et al. [118] went a step further toward screening, embedding multiple oral cell types in a natural-polymer matrix made from natural polymers (collagen type I and hyaluronic acid) crosslinked by blood-coagulating factors. They identified plasminogen activator inhibitor-2 (SERPINB2) as a sensitive toxicity marker and coupled it to a fluorescence readout, arguing that these chips can be tuned into quantitative decision tools rather than qualitative proxies.

In a complementary study, a customized organ-on-a-chip microfluidic device was fabricated for the dynamic culture of oral mucosa [119]. The open-type platform featured two interconnected chambers for long-term perfusion and was characterized with respect to additive manufacturing (AM) parameters, as well as its mechanical and biological performance. A split-inlet channel architecture ensured uniform medium distribution and symmetric flow velocities across the culture area. This 3D system can be further scaled to a high-throughput format and used to evaluate the biocompatibility of biomaterials or drugs that contact the oral mucosa.

Complementing those efforts, Hu et al.’s [120] “gum-on-a-chip” brought immune and microbial variables into scope, using a biomimetic gingival tissue to show how Porphyromonas gingivalis suppresses immune-cell recruitment and perturbs CD14 signaling—an application-oriented demonstration that these systems can surface actionable host–pathogen phenotypes relevant to periodontal disease modeling and early drug discovery.

#### 6.1.7. Renal-on-A Chip

Guimarães et al. [21] offer a decidedly application-oriented renal proximal tubule-on-a-chip in which a collagen type I–grafted PDMS microchannel enables fast, flow-ready adhesion of renal proximal tubule epithelial and human umbilical vein endothelial cells within 30 min, so the device can actually be used to run assays rather than wait days for stabilization. In this configuration, the chip could perform functional readouts that are important for preclinical decision-making—size-selective barrier behavior, albumin uptake under perfusion, glucose reduction toward physiological levels, and a clear nephrotoxic response to amphotericin B—while operating at near-physiologic shear. The immediate implication for the design implying collagen as a barrier interface in kidney chips is straightforward but important: the chemistry of how collagen type I is tethered to PDMS sets not only adhesion kinetics but also experimental uptime and data quality, effectively turning contact angle and fibril presentation into experimental variables that should be reported alongside permeability or viability.

Lapin et al. [121] built a perfused kidney-on-a-chip that sits tubular epithelia inside collagen type I microtubular channels and, crucially, lets the experimenter decouple wall shear from intraluminal pressure. In this setting, PKD1-deficient proximal cells dilated mainly via over-proliferation regardless of the hydrodynamic regime, whereas PKD1-deficient collecting-duct cells showed a very different, pressure-sensitive phenotype: flow alone muted dilation, but adding ~10 mbar intraluminal pressure restored it—an effect that could be blunted simply by stiffening the collagen type I gel (6 → 9 mg/mL). Functionally, this moves renal chips from proof-of-transport studies toward disease-mechanics assays that can rank the dominant physical drivers of pathology, including shear, pressure, and matrix stiffness. For collagen type I-based barrier interfaces, the immediate lessons are practical: (i) report and control intraluminal pressure (not just flow); (ii) explicitly tune collagen concentration and fibrillogenesis kinetics; (iii) stratify endpoints by nephron segment, as proximal and distal epithelia exhibit distinct failure modes.

### 6.2. Cancer-on-Chip and Metastasis Studies

Collagen type I remains the standard ECM scaffold for microfluidic models that interrogate metastatic escape, largely because it provides a tunable, fibrillar barrier through which malignant cells must negotiate to reach perfused vessels. To survive and proliferate, cancer cells remodel their phenotype to match the microenvironment. On conventional plastic, this includes reprogramming focal adhesion gene expression, metabolic state, and differentiation behavior. Without adaptation, cancer cells may die. These culture-induced changes frequently shift therapeutic sensitivity and drug response profiles. Recent experimental studies illustrate how this collagen-defined interface is being leveraged not only to visualize invasion and intravasation, but also to pressure-test anti-metastatic strategies and organotropism hypotheses [122].

Tan and colleagues [123] built a breast cancer “invasion-toward-a-microvessel” platform in which MDA-MB-231 cells advance through a fibrillar collagen type I gel toward a neighboring, lumenized HUVECs channel. The key finding is that endothelial cells reprogram tumor metabolism, increasing glycolysis and oxygen consumption, which in turn directs invasion toward blood vessels. Reducing glucose availability or inhibiting adenosine triphosphate synthase diminished endothelial cell-driven invasion. With the collagen type I gel serving as the only path between tumor and vasculature, the work establishes a direct connection between metabolic crosstalk and barrier crossing. For metastasis, the barrier is not passive but metabolically active, and nutrient availability becomes practical for modulating invasion in collagen-rich stroma.

Ozer et al. [124] pushed the same basic architecture into screening territory by adopting an OrganoPlate^®^ 3-lane design where the central path is a 5 mg/mL rat-tail collagen type I gel that separates a tumor/fibroblast compartment from a perfused HUVECs microvessel. The team ran a targeted small-molecule library and, critically, scored both invasion through collagen and intravasation across endothelium. Co-culture or vascular endothelial growth factor (VEGF) depressed endothelial resistance at 48 h (20%), whereas 5 µM Imatinib held TEER steady. At the interface, barrier stabilization—reflected in higher TEER—was accompanied by reduced transendothelial entry, and notably this occurred without any measurable reduction in migration through collagen. The collagen type I lane functionally partitions matrix invasion from endothelial passage, delivering the decoupling that anti-metastatic studies require. Equally important, the workflow is compatible with high-throughput screening, which should facilitate wider use.

**Figure 23:**
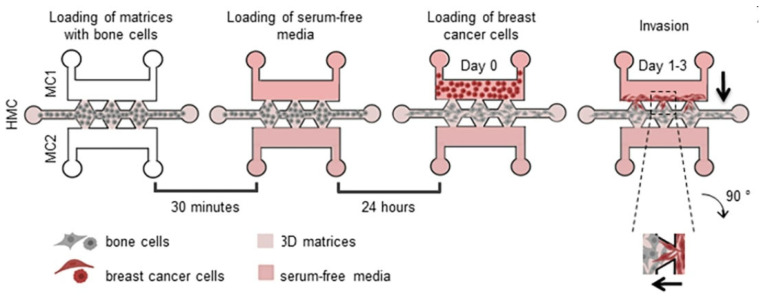
Schematic representation of the invasion assay on IC-Chip: a zoom-in inset of the imaging area between two posts (**right**). Arrows indicate the direction of invasion. Reproduced in part with permission from [53].

Firatligil-Yildirir and co-workers [53] created paired invasion/chemotaxis and extravasation chip models for breast cancer bone metastasis. In this configuration, the bone-side microenvironment channels were loaded with 3 mg/mL collagen type I, either alone or in combination with chitosan or agarose, to embed osteoblasts, bone-marrow stromal cells, and monocytes (Figure 23).

Two points are salient: (i) collagen type I better reproduced a bone-like environment for bone cells than basement-membrane substitutes, both in cellular behavior and in mechanics; (ii) the platforms resolved distinct invasion/extravasation phenotypes for bone-tropic (BoM-1833) versus lung-tropic (MDA-MB-231) cells that track with in vivo outcomes. Recognizing that stiffness changes over time in culture, the authors employed in situ, contactless rheology under physiological culture conditions and found that cell-dependent matrix stiffening coincided with altered invasion through type I collagen. These findings are directly translatable to metastasis-on-chip engineering: accurate modeling of bone homing requires explicit control of collagen type I composition and viscoelastic evolution, as these parameters define the cellular decision landscape.

Chen et al. [125] developed an ovarian tumor-on-chip that recapitulates the ECM milieu and ascitic fluid flow characteristic of ovarian cancer. The device underscores the key role of basement-membrane proteins—collagen, and laminin—in regulating cell migration. Collectively, these findings highlight the need to account for ECM composition and its synergy with flow-induced shear stress when studying cell migration and, more broadly, when engineering tumor- microenvironments-on-a-chip systems.

Wu et al. [126] reported a matrix-free, biomimetic 3D microfluidic platform that generates cell spheroids and subsequently embeds them in collagen type I to recapitulate the tumor microenvironment, enabling assessment of how collagen influences doxorubicin-induced drug resistance in MDA-MB-231 cells. Compared with conventional two-dimensional cultures, this 3D chip more faithfully captures in vivo conditions and provides a practical workflow for spheroid formation and manipulation, supporting investigations into fundamental tumor cell physiology and phenotype responses.

Finally, Ikeda et al. [127] engineered a tumor-microvessel on-a-chip that positions colorectal tumor organoids at defined distances from a perfused microvessel embedded in collagen gel. The near-contact configuration reveals an unambiguous, sequential intravasation mechanism: collective migration along the collagen-vessel interface, vessel co-option, and the release of tumor-cell clusters into the lumen. Mechanistically, endothelial transforming growth factor β (TGF-β)/activin signaling and features of endothelial-to-mesenchymal transition were necessary for cluster release. Two immediate implications emerge: (i) the collagen type I matrix functions as more than a passive resistor, actively organizing cluster–vessel contacts; (ii) interventions directed at endothelial-to-mesenchymal transition or TGF-β–family cues could be assessed using platforms that accurately reproduce the collagen–endothelium junction where clusters form and traverse.

### 6.3. Context-of-Use and Validation Status of Collagen Type I Microfluidic Barriers

To orient the reader, the following subsection distills where collagen type I barrier interfaces in microfluidic models appear closest to clinical or regulatory relevance.

First, gut-barrier models using collagen type I gels in high-throughput formats (e.g., OrganoPlate) are already used for toxicology-style readouts (i.e., TEER, permeability, and cytotoxicity) with clear, dose–response behavior to reference enterotoxins. In this platform, Caco-2 tubules are seeded against collagen type I, and the outputs align with translational endpoints of regulatory interest (e.g., barrier integrity and inflammation markers) [91].

Second, collagen type I-based skin barrier chips seem to mature quickly. A microfluidic, innervated epidermis-on-chip that uses collagen type I hydrogels to build an acellular dermal ECM and to support a stable, ALI-differentiated epidermal layer demonstrated robust barrier formation and functional readouts (FITC-dextran permeability; stratification markers) [107]. Given the long regulatory history of reconstructed human epidermis tests, these collagen-enabled skin chips seem well-positioned for targeted, context-of-use claims (e.g., mechanistic irritation or neuropathic endpoints) once inter-lab reproducibility is nailed down.

Third, in the respiratory space, a collagen tube-based airway-on-chip recreated a perfusable epithelial conduit with extended viability and physiologic behavior [85]. While not yet tied to a specific regulatory test guideline, the combination of native collagen type I architecture and airway-relevant exposures (aerosols, ALI) could suggest plausible near-term uptake for comparative performance studies (e.g., mucociliary endpoints or irritancy).

Fourth, BBB models using collagen type I microfibers to guide capillary network self-assembly have recently shown improved microvascular organization and astrocyte survival (via β1-integrin signaling) in a microphysiological BBB system [103]. These results are highly encouraging for drug-delivery applications and neurovascular disease research. However, regulatory convergence will take longer because, unlike hepatic or dermal systems, the central nervous system barrier has a limited set of recognized qualification exemplars and an underdeveloped compendium of reference compounds.

### 6.4. Per-Device Cost and Scalability

Across recent experimental studies, explicit “price tags” per device remain uncommon. However, several studies employing collagen type I at barrier interfaces describe manufacturing decisions that influence both cost and throughput. Three recurring patterns were observed: (i) open, plate-style formats that spread handling effort across many chips; (ii) cleanroom-free fabrication—such as plotter cutting (xurography) or desktop 3D printing—using commodity consumables; (iii) membrane-free designs that cast or pattern collagen type I directly, thereby reducing part count and assembly steps.

First, plate-integrated chips that culture epithelium against collagen type I illustrate a practical trade-off in which throughput could reduce effective unit cost. Using a membrane-free OrganoPlate, Morelli et al. [91] conducted 64 parallel gut-tubules assays pre-seeded on collagen type I, highlighting the 384-well plate geometry and integrated TEER capability. While they do not quote a per-chip price, the compact layout concentrates hands-on time and consumables, improving cost per data point.

Second, desktop-fabricated molds for open microfluidics support collagen type I hydrogels and enable credible cost benchmarking. O’Brien et al. [48] used 3D-printed molds to form open channels in collagen (2–4 mg/mL), reporting materials costs of USD 1–2.50 per sample, compared to ~USD 55 per sample for soft-lithographic fabrication (not including cleanroom/machine overhead). These values provide a realistic materials-cost floor when the collagen interface is molded rather than confined between bonded layers.

Third, cleanroom-free xurography provides a practical route to reduce both capital and iteration costs for microvessel MPSs embedded in collagen. In Agarwal et al. [128], modular PDMS devices were cut on a benchtop plotter and used to culture endothelial microvessels in user-defined ECMs (including collagen type I). The authors emphasize sub-$1000 capital requirements and fast prototyping, which typically dominate early unit economics during laboratory scale-up.

A complementary route to reduce parts count is to replace synthetic membranes with collagen itself. Gao et al. [85] engineered self-supporting collagen tubular lumens for an airway barrier—where the tube itself serves as the barrier—eliminating membrane sourcing and bonding. Although no cost analysis is provided, the bill of materials is visibly simpler and relies on readily available collagen solutions.

Relatedly, viscous-finger patterning of collagen type I supports in situ endothelialized lumen formation, apparently without the need for sacrificial templates or inserts—streamlining fabrication. In recent demonstrations [129], a lower-viscosity stream displaces the collagen type I solution inside the device to define the lumen, producing a membrane-free barrier surface that is readily replicated and well suited to batch processing.

Thermoplastic platforms are critical for scaling beyond prototyping. In Olaizola-Rodrigo et al. [72], pillarless COC/COP chips leverage plasma patterning to confine hydrogels, collagen-coat the endothelial channels, and validate BBB performance with a hydrogel core. Since COC/COP are compatible with injection molding and plotter cutting, they could enable mass-manufacturing workflows once the geometry is fixed, thereby lowering per-unit costs relative to PDMS.

On the ‘vascular bed around tissue’ dimension, platforms that introduce organoids/spheroids into microchannels and promote network formation within ECM increasingly emphasize simplified loading and parallelization. While matrix choices vary across studies, numerous recent implementations retain collagen (as a surface coat or gel phase) and demonstrate 10-channel layouts, open-top accessibility, or sequential loading—reducing handling time per construct and thereby labor costs. For instance, an open-top vascular chip that supports two interconnected 3D vascular beds was explicitly configured for sequential loading and parallel perfusion [87]. Although the hydrogel formulation is flexible, the open architecture has direct cost implications when collagen is used as the barrier ECM.

Beyond gut and airway models, BBB work using collagen-based barrier interfaces is converging on practical manufacturing details. In Nakayama-Kitamura et al. [103], collagen type I microfibers stabilized the BBB microvasculature, improving network robustness and thereby reducing failure-driven scrap. Parallel efforts implement BBBs in pillarless and plate-based devices with collagen-coated endothelial channels. While explicit pricing is absent, these strategies concretely simplify assembly and migrate the platform to scalable substrates such as COC/COP.

Finally, oral and skin barrier models keep collagen at the tissue interface and increasingly adopt additive manufacturing for chassis fabrication. A recent oral-on-chip employed a collagen–hyaluronic acid matrix cast within PDMS device produced from PLA 3D-printed molds [118], using tools and materials common to most academic laboratories—a strong indicator of low per-unit cost and facile replication. In parallel, full-thickness, perfused skin-on-chip devices built via high-precision micro-stereolithography emphasize consistent part quality and multi-chip holders that enable parallelization and typically lower cost per sample, all while preserving collagen-rich dermal equivalents.

To summarize, the per-device cost outlook for collagen type I barrier interfaces is trending positively—driven by cleanroom-free patterning (e.g., xurography, 3D-printed molds), membrane-free collagen lumens/tubes, and the use of thermoplastics for scalable production. What the field now needs is consistent reporting of materials cost and cycle time, so methods can be compared on a level playing field. Adding a brief “cost and scale” paragraph in the ‘Materials and Methods’ section would make this immediately actionable for both end-users and reviewers.

## 7. Limitations and Challenges

Collagen type I remains the default choice for building biological barrier interfaces in microfluidic systems, largely because it self-assembles under benign conditions, supports a wide range of cell phenotypes, and can be remodeled by resident cells. That said, several practical limitations continue to surface once these constructs move from proof-of-concept devices to longer, more demanding experiments.

(i) Mechanical fragility and time-dependent degradation: Freshly polymerized collagen gels are mechanically soft and often brittle at low collagen concentrations—the very regime used to preserve cell viability and diffusion. Even when initial handling is feasible, gels can relax and compact over days, driven by thermal fluctuations and cell-mediated remodeling. The consequence is a slow drift in barrier thickness and porosity that translates into evolving permeability profiles [17,35]. Enzymatic degradation (e.g., endogenous matrix metalloproteinase - MMP activity) further complicates long-term operation, especially in inflammatory or tumor-mimetic cultures [130]. Practical countermeasures—slightly higher collagen content, gentle confinement with posts or porous supports, or low-dose MMP inhibition—hold the line only up to a point. Past a week or two of continuous perfusion, many devices exhibit measurable softening and geometric changes that are hard to ignore [17].

(ii) Batch-to-batch variability: Collagen sourced from different lots (or vendors) frequently yields gels with different fiber diameters, crosslink densities, and pH-dependent assembly kinetics. Those microscopic differences show up macroscopically as shifts in storage modulus and apparent permeability for the “same” percent gel [131]. The problem is most acute when the barrier must sit at a narrow mechanical set point [132], for instance, to preserve endothelial tight junctions [94]. Routine rheology and fibrillogenesis assays can mitigate unanticipated variability, but they add cost and rarely eliminate it. In practice, many groups still re-tune polymerization conditions with each new lot—a habit that is not scalable.

(iii) Barrier tightness and tuning permeability: Achieving physiological relevant tightness is nontrivial because transport across collagen-based barriers is steered by two moving targets: the gel’s fibrillar mesh and the cells that line or populate it. The familiar tuning variables—collagen concentration, assembly pH/temperature, ionic strength, and polymerization time—indeed shift pore size and tortuosity, but they simultaneously reshape cell adhesion, spreading, and matrix remodeling. With perfusion the system becomes fully dynamic: shear can reinforce tight junctions in certain epithelia even as it accelerates collagen compaction [133]. In practice, many studies lean on short-term TEER or tracer readouts to claim barrier integrity [91], only to observe rising leakage as the matrix compacts or is remodeled over time [130]. One should mention that across collagen type I-based microfluidic barriers, TEER is most informative when interpreted against the platform’s own baseline and paired with functional endpoints. Depending on electrode geometry and distance to the monolayer, hydrogel conductivity, cellular composition, flow, and tubular vs. planar architecture, absolute values can deviate from Transwell conventions. Studies presented in this review illustrate this clearly—from micro-positioned TEER in a human BBB model and MP-ECIS that remains sensitive in hydrogels, to gut and metastasis systems where TEER co-varies with permeability, cytotoxicity, or invasion. As a general observation, in vitro TEER values—especially on collagen type I barrier interfaces—tend to be lower than in vivo owing but not being limited to: limited cellular maturity/density and junctional development, ECM composition and mechanics, electrical shunts through hydrated matrices or imperfect sealing, geometry/edge effects, and area normalization in microchannels, and non-physiological culture conditions (temperature, ionic strength, and shear). Therefore, since absolute TEER from an OoC rarely maps 1:1 onto “Transwell conventions,” we provided within this review concise, study-specific guidance in lieu of global physiological ranges, which could be misleading for these collagen-integrated, perfused geometries.

A sturdier strategy combines passive control of the matrix formulation with active stabilization—supporting cell–cell junctions, applying modest shear, and, where appropriate, introducing low-dose crosslinking once polarity is established.

(iv) Crosslinking strategies: Chemical and enzymatic crosslinkers—EDC/NHS, genipin, and a range of photo-mediated systems—are routinely used to stiffen collagen type I gels and slow their enzymatic degradation [29,63,134]. Stronger crosslinking typically compacts the fibrillar mesh and lowers diffusivity, which can throttle nutrient transport unless perfusion is increased [29]. Some chemistries or their by-products compromise cell health or reduce effective adhesivity by altering available motifs; for example, high EDC/NHS loads in collagen hydrogels reduced MSCs viability in 3D culture [134]. Photo-crosslinking affords spatial control, but the light/initiator pair must be chosen carefully because irradiation and radical chemistry can depress viability or generate reactive species [29]. A pragmatic pattern has therefore emerged: applying the lightest crosslinking needed to keep mechanics stable over the experimental window, and if adhesion drops, restoring bioactivity by supplementing specific ligands or blending a modest fraction of native ECM proteins [63,134]. Even then, heavily crosslinked gels tend to resist cell-driven remodeling—useful when a barrier has to stay tight, but counterproductive when remodeling is the readout [134].

(v) Integration and bonding with synthetic materials: Hybrid devices have to couple a collagen interface to PDMS, thermoplastics, or glass without wicking, delamination, or bypass flow [17,135]. Wet-to-wet bonding on hydrophilic plastics is especially unpredictable; plasma-activated surfaces drift back toward their native state, and some adhesion-promoting chemistries change what cells “see” at the interface by grabbing proteins or biasing attachment [17,72]. Mechanical anchoring—microposts, undercuts, or interlocking features—do make the construct harder to peel apart, yet they complicate tooling and redistribute stresses so that failures start at corners or constrictions; moving to pillar-less confinement was shown to avoid such local shear/stress distortions while maintaining gel pattern fidelity [72]. Finally, collagen gels and the surrounding polymers rarely “age” mechanically at the same pace during operation: even modest perfusion or cycling loading can nudge geometries over time via gel compaction or partial debonding at the gel–wall interface—effects captured by time-lapse measurements of collagen immobilization/detachment on COP devices and by pillar-free designs that deliberately remove stress-concentrating posts to maintain gel confinement [17,72].

(vi) Scalability and standardization: Most collagen type I barriers are still assembled by hand—great for prototyping, but poor for reproducibility. Subtle shifts in neutralization strategy, pre-gel temperature, or the delay between mixing and casting can nudge fibrillogenesis and produce discernible changes in fiber orientation and network texture [73,136]. When devices are fabricated in batches, spatial non-uniformities can creep in during curing—edge-to-center gradients driven by heat flow and local evaporation that bias gelation kinetics and, later, mass-transfer profiles. Standard operating procedures help, with groups reporting keeping components on ice, rotating chips during early polymerization, and maintaining humidified reservoirs to blunt evaporation, all of which temper gradients across the casting field [72]. Standard operating procedures and tooling—pre-cooled pipettes, simple jigs to shorten dwell times—do push variance down, yet cross-site comparability is still modest because each laboratory group tends to “tune” collagen differently [73]. From a translational standpoint, the lack of reference materials and calibration routines (e.g., permeability or shear-modulus readouts) and, critically, portable quality-control metrics that can travel with a device: recent experimental papers quantify contraction, confinement under flow, and diffusion-through-gel as study-specific surrogates rather than standardized benchmarks, which complicates multi-lab comparisons and automated manufacturing pipelines [17].

(vii) Optical compatibility: Fibrillar collagen does scatters light, and once the matrix is compacted or chemically crosslinked the problem worsens—what looks like a minor tweak in matrix chemistry or packing can quickly degrade contrast for high-numerical-aperture imaging and throw off quantitative fluorescence. In practice, groups working on collagen-based vascular chips report that conventional widefield or standard confocal often underperforms in these gels [137]. However, some mitigations do exist. Optical workarounds—clearing after fixation or simply casting thinner collagen type I barriers—do improve image quality and depth; yet the same modifications can perturb the very transport properties of interest (i.e., diffusion paths, hydraulic permeability), so the trade-off resurfaces when one tries to benchmark function across devices and laboratories [80,137].

(viii) Long-term biochemical stability: Serum proteins and cell-secreted factors readily adsorb to collagen and can be retained or released slowly from the matrix, which introduces hysteresis into exposure profiles and make nominal “washout” experiments look cleaner on paper than they are on practice [90,138]. Neither effect is catastrophic, but both can bias interpretation unless explicitly measured and controlled during on-chip dosing and clearance steps [90].

(ix) Multilayer collagen construction: Building physiologically layered barriers is attractive, but their stability hinges on managing fragile interfaces. In tri-culture formats where a collagen type I hydrogel carrying fibroblasts sits between an apical epithelial ALI and a basal endothelium, the architecture is surprisingly sensitive to order—cells must be introduced in a defined order—a reminder that the realism of multilayer stacking comes with the liability of interface control [81]. Gut-on-chip platforms echo this theme: epithelial and microvascular layers can operate stably under peristaltic-like actuation, yet performance is ultimately gated by the gel compartment. Measures of barrier integrity and transcriptional responses track the collagen’s capacity to maintain epithelial polarity and endothelial viability under load [139]—hence the need to report matrix thickness and composition alongside the biological readouts. The pattern holds in inflammation models. In a composite system with a collagen–alginate lamina propria populated by fibroblasts beneath an epithelial sheet and above an endothelium, the formulation can be tuned to preserve fibroblast metabolism and morphology [140]. However, gel composition and thickness still set the window for co-culture longevity and for transport data to remain interpretable. A similar trade-off appears in BBB-on-chip tri-cultures, where astrocytes and pericytes embedded in a collagen middle ECM sit next to a perfused endothelial channel: the geometry works as designed, but ECM identity and layer spacing end up governing both the fidelity of cell–cell crosstalk and the comparability of barrier metrics across platforms [141]. In practice, the sturdier path pairs careful matrix specification—composition, thickness, spacing—with active interface management: seeding sequences that lock in polarity, gentle perfusion to stabilize contacts, and, when appropriate, modest reinforcement of the ECM once the layers are in place.

Collectively, these points clarify why collagen type I barrier interfaces remain a compelling choice for biomimetic microfluidics and why they still call for careful engineering to perform consistently across the time scales and perturbations typical of modern assays.

## 8. Conclusions

Recent literature reports confirm that collagen type I remains one of the most versatile and biologically faithful materials for building barrier interfaces in microfluidic systems. Its fibrillar architecture, cell-adhesive ligands, and enzyme-responsiveness allow epithelial, endothelial, and stromal compartments to communicate in ways that synthetic membranes still struggle to capture. Collagen type I supports contact guidance, matrix remodeling, and polarized transport, and it tolerates a wide range of biochemical functionalization. In short, if the goal is to recapitulate the instructive complexity of native extracellular matrix while retaining experimental control, collagen type I stands as the pragmatic starting point.

At the same time, the field has become more pragmatic about the engineering compromises involved. The recently reviewed literature (i.e., 2023–2025) shows a clear shift toward ultra-thin collagen type I barriers—often tens of micrometers or less—to improve diffusive exchange and short-range signaling. These thinner constructs bring obvious benefits for kinetics and sensitivity, but they also magnify problems of handling, compaction, and delamination under shear. In this context, several solutions are reaching maturity: gentle crosslinking strategies that preserve bioactivity, hybrid interpenetrating networks, and increasingly precise patterning/bioprinting that anchors barrier interfaces directly to device features. Collectively, these advances make it realistic to tailor collagen’s microstructure—fiber alignment, pore size, and local stiffness—to the biology under study, instead of accepting the gel outcome dictated by the baseline protocol.

Methodologically, the community would benefit from common reporting standards—collagen source, fibrillogenesis conditions, fiber metrics, residual crosslinker, and in situ mechanics—not just nominal concentration. Controlling microstructure at scale (i.e., alignment, gradient architectures, and anisotropic stiffness) is a near-term opportunity, as is integration of collagen type I barrier interfaces with embedded sensors (miniaturized transepithelial/transendothelial electrical resistance, oxygen, and pH) for continuous monitoring. Long-lived cultures will require strategies to limit compaction and drift without blunting physiologic remodeling. On the biological side, immune-competent co-cultures, vascularized interfaces, and patient-derived matrices should be prioritized, especially where disease phenotypes are matrix-led. Finally, interoperable data and open protocols—paired with image-based quality control of fiber architecture—would greatly improve cross-lab comparability.

In conclusion, collagen type I has earned its status as a cornerstone material for biomimetic barrier interfaces in microfluidic devices. The recent trend toward thinner, better-controlled barrier interfaces is encouraging, provided structural integrity and manufacturability are treated as design constraints not relegated to secondary considerations. With measured standardization, judicious hybridization, and genuinely interdisciplinary collaboration, collagen type I-based barrier interfaces are well positioned to move from elegant models to decision-support tools and, ultimately, to translational applications where fidelity of barrier function is of paramount importance.

## Figures and Tables

**Table 1:** Structural and Functional Comparison of Synthetic versus Biological Collagen Type I Barrier Interfaces in Biomimetic Microfluidic Systems.

Feature	Synthetic/Hybrid * Collagen Type I Barrier Interfaces	Ref.	Biological (Native/Reconstituted) Collagen Type I Barrier Interfaces	Ref.
Biochemical cues	Require functionalization (e.g., with PLLA, PLGA, PCL, or PET nanofibers) to improve adhesion and biochemical signaling	[36,37]	Provide extracellular support that mimics the biochemical and mechanical properties of ECM, enabling direct cell–ECM interactions and supporting cell adhesion, growth, and migration; ECM–barrier biochemical coupling	[17,35,38]
	Limited integrin-binding motifs without additional surface modification	[36]	Native fibrillar motifs, integrin-binding sites, growth-factor sequestration; strong crosstalk with other ECM proteins (e.g., fibronectin) affecting endothelialization	[7]
Mechanical properties **	Mechanical properties modulated via crosslinking and polymer hybridization	[19,27]	Cell-remodeled stiffness and viscoelastic drift over culture time; fiber alignment under flow or contraction, leading to evolving matrix mechanics	[39,40]
	Independently tunable stiffness/viscoelasticity via double networks or porous architectures; engineered stress-relaxation.	[41,42]	Limited mechanical properties and stability	[36]
Permeability **	Permeability tailored by fiber diameter, porosity, and crosslinking	[36]	Sensitive to gel concentration, polymerization conditions, and enzymatic remodeling	[43]
	Lower pore volume and diffusivity after crosslinking due to nanofiber densification	[36]	Permeability changes quantified via TEER/marker flux in epithelial/endothelial barriers	[38,43,44]
	PEG/collagen hydrogels maintain network integrity under culture while enabling nutrient exchange	[42]		
Biodegradability	Overall degradation rate slowed via crosslinking (e.g., GTA or EDC/NHS) or decoupled via synthetic network (e.g., PEG) to provide stability during long assays	[27,45,46]	Rapid degradation rates	[15]
	Degradation directly linked to functional stiffness shifts; controlled degradability under culture	[42,47]	Intrinsically enzymatically degradable (MMP/collagenase); degradation links directly to barrier loss and increased transmural transport	[35,38]
	Degradation rates tunable from hours to weeks	[27]		
Biomimicry	Mimic native collagen partially	[36]	High structural and functional biomimicry	[23]
	Can simulate more biophysical conditions in the physiological and pathological microenvironment	[19]	Reproduction of in vivo-like migration patterns and stromal interactions	[40]
	Geometry and fiber-like architecture replicated by microfabrication/3D printing; composites (agarose–collagen, chitosan–collagen) capture fibrillar cues while keeping macro-shape/porosity stable	[48,49]	Closest biochemical fidelity to stromal ECM; supports native cell phenotypes in organ-specific barriers (e.g., collagen-based membranes in gut-on-chip)	[7]
Applications	Integrated in hybrid OoC systems (e.g., PET/PDMS nanofiber barrier interfaces) for cell adhesion studies, flow-induced response assays, and nanoparticle exposure under dynamic conditions	[50]	Disease-mechanism studies where ECM remodeling is central (e.g., inflammation- or pathogen-driven barrier failure), and assays probing matrix–cell feedback	[35]
	High-throughput, long-term organ-on-chip barrier models (gut/BBB/placenta) where stability and controlled mechanics are critical; platforms for drug transport studies	[42,51,52]	Drug transport across collagen-coated/ECM-conditioned barriers	[44]
			Collagen type I matrices as the stromal barrier for metastasis-on-chip studies	[53]

* Many synthetic collagen barrier interfaces described in the literature are hybrid composites combining collagen type I with synthetic polymers (e.g., PLLA, PCL, or PET) or crosslinking agents. ** Quantitative values (e.g., permeability coefficients, tensile strength) should be interpreted in the context of the original publications; table entries are summarized interpretations of reported findings.

**Table 2:** Crosslinker selection guide: practical criteria for selecting a collagen type I crosslinking strategy in biomimetic microfluidic systems.

Crosslinker/Method	Typical Use Case	Stiffness Control	Cytotoxicity Tolerance	Cost/Complexity	Reversibility	Ref.
Genipin (amine crosslinker)	Cell culture substrates to study effects of ECM stiffness (mimic brain, lung, liver, tumor tissues)	Young’s modulus tuned from 0.0292 to 12.5 kPa by 0–10 mM genipin	Low at ≤0.3–0.6 mM; ≤0.5–1 mM often tolerated; higher doses (≥1–2 mM) become toxic	Simple mix-and-gel (inexpensive)	No	[63,64]
EDC/EDC+NHS (direct addition); EDC/EDC+NHS (immersion)	Tissue engineering	EDC/NHS (direct addition)—lowest Young’s modulus, tensile strength, and braking force; most flexible; EDC/NHS (immersion)—lower Young’s modulus; highly durable	NA	Moderate (aqueous; protocol-sensitive)	No	[65]
GTA cross-linking to collagen and PLL coating	Microfluidic immobilized enzyme reactor for drug metabolism studies and multi-enzyme platform	NA	NA	Cheap, easy to fabricate and adaptable chip structure	No	[66]
Single functionalization of atelocollagen with 4-vinylbenzyl chloride (4VBC); UV-cured	Guided bone regeneration membranes	Higher suture retention strength	NA	Cost-effectiveness	No	[30]
Sequential functionalization with 4VBC then methacrylic anhydride (MA); UV-cured	Guided bone regeneration membranes	Two-fold increase in compression modulus; two-fold increase in median (interquartile range) elastic modulus	NA	Cost-effectiveness	No	[30]

Note: This table is intended as a practical guide; final selection should consider device geometry, shear exposure, sterilization workflow, and any assay-specific constraints.

**Table 3:** Side-by-side comparison of the strategies for integrating collagen into microfluidic platforms discussed in this section. The concise ratings were distilled directly from the cited studies (see the table’s final column).

Methods of Integrating Collagen into Microfluidic Platforms	Ease of Fabrication	Biological Fidelity	Mechanical Robustness	Cost	Specific Applications	Ref.
Collagen nanofiber membrane sandwiched between PDMS layers	Easy—oxygen plasma treatment; standard PDMS workflow	Good—HUVECs/C6 co-culture under flow	Good—leak-free; stable at 10 µL/min	Low—simple PDMS-based microfluidic device	Rapid test for drug screening, permeability, cell viability measurements, and disease modeling	[19]
Collagen-coated PET insert in injection-molded poly-carbonate chip	Moderate—mass-production with injection molding	High—HRPTECs–HUVECs co-culture; TEER/permeability readouts	High—stable under shear conditions for ~14 days	Reduced	Nephrotoxicity modeling with readouts	[68]
Cross-linked electrospun collagen scaffold sandwiched between laser-cut Teflon membranes in a 3D-printed housing	Moderate—no cleanroom	High—robust transwell	High—leak-free; 7-day perfusion	Low–Medium—parts printed easily	Transport assays	[69]
Open-microfluidic channels molded directly in collagen using 3D-printed forms	Easy—simpler to manufacture than traditional closed microfluidic cell culture systems; minimal specialized equipment	Good/Moderate—HUVECs vessel mimics; high cell viability (>89%); hypoxia-responsive CD31	Good/Moderate—channels faithful to molds down to 400 µm	Low—materials ≈ $1–2.50 per sample (depending on the type of 3D printing technology used to fabricate the molds)	Open blood vessel mimics; hypoxia/oxygen response assays	[48]
Serially patterned collagen zones along a perfusable microchannel (needle-molded lumen)	Easy—standard benchtop equipment; avoids photolithography	Good—spatial control of cell–cell interactions; localized epithelial sprouting with stromal patterning	High—parabolic flow profile with no disruptions; consistent diameters; robust microchannels	Low—simple, inexpensive methodology	Interrogation of vascular function and endothelium	[70]
Viscous-finger patterned collagen lumen with sequential seeding (VoC)	Moderate—PDMS soft-lithography; viscous-finger lumen; perfusion setup	High—hiPSC-EC vessels + embedded macrophages; whole-blood perfusion readouts	High—stable under whole-blood perfusion; thrombus assays	Medium—standard microfluidics + perfusion hardware	Establishing protocols for viscous finger patterning	[71]
Pillarless thermoplastic chip with plasma-defined hydrogel confinement (collagen/fibrin)	Moderate—injection-molded parts; oxygen-plasma bonding; simpler than photopatterning	High—BBB model with human astrocytes/pericytes; ~100% survival rate (7 days)	High—hydrogel reliably confined; diffusion/permeability validated (day 7)	Medium—tooling/equipment implied	BBB-assembly in the pillarless microfluidic device	[72]
Microfluidically aligned collagen fibers on dishes (micropillar array designs)	Easy—easy-to-use piggyback platform; easy to set up and run	Good—tenocyte elongation; expression of tenocyte markers	Good—fibrillar collagen confirmed (D-banding of 65 nm)	Medium—standard PDMS/UV photolithography	Tendon repair/regeneration	[73]
STOMP: open microfluidics and capillary pinning to pattern free-standing tissues	Easy—open-to-air channels; simple pipetting; removable patterning device	Good/Moderate—multi-region suspended tissues; diseased-healthy boundaries; tissue-type interfaces	Good/Moderate—free-standing constructs via capillary pinning (qualitative)	Low—3D-printed parts; surface-tension driven patterning	Interfacial tissue modelling; suspended tissues with precise patterning; dynamic and spatially controlled constructs	[74]
COP-based microfluidic devices with surface plasma activation, APTES, or PAA-PG for covalent bonding	Moderate—UV initiated single-step photografting; silanization workflow on thermoplastics	High—long-term 3D culture of contractile cells; necrotic core model	High—PAA-PG treatment for structure preservation (90% area, 8 days)	Medium—additional reagents (UV, initiators)	Co-culture models of angiogenesis, wound healing, tumour microenvironment and ischaemia	[17]
PDMS devices activated by O_2_ plasma then coated with PDA to enhance adhesion of collagen type I hydrogels	Moderate—standard plasma activation; PDA coating	High—sustained PDAC 3D tumor spheroids	High—stronger adhesion & stability of collagen gels; 7–11-day cultures	Medium—PDA chemistry	Tumor-on-chip	[60]
Air-plasma pre-treated PDMS microchambers followed by collagen coating (single-step activation integrated at sealing)	Easy—single-step air plasma activation; collagen coating	Good/Moderate—MSC growth to confluency in 5 days	Good/Moderate—stable hydrophilization; PDMS-collagen composite layer (7 days)	Low/Moderate—air-plasma; culture reagents	Organ-/bone-marrow-on-chip	[75]
Collagen/MWCNTs nanocomposite coatings (+ GTA crosslinking) evaluated under shear stress flow	Moderate—nanomaterial dispersion; substrate treatment; coating/GTA crosslinking	Good/Moderate—reduced thrombogenicity compared with titanium	High—high resistance to shear stress flow; GTA crosslinking increases stability (~2 times)	Medium—nanomaterials and instrumentation	Hemodynamics	[76]
Argon-plasma activated, microstructured PDMS patterns grafted with collagen type I for guided cell culture	Moderate—photolithography + argon plasma activation + coating	High—excellent myoblast alignment; cytocompatible surface	Good—durable collagen grafting on plasma-activated PDMS microstructures	Medium—microfabrication steps	Myoblast guidance; muscle-relevant tissue engineering	[77]
Collagen type I + BG NPs loaded into PDMS chip with trapezoidal posts (surface-tension gel loading)	Moderate—PDMS, soft-lithography, oxygen-plasma bonding	High—high viability (L929 cells); optimal composite selection for microenvironment mimicry	High—BG NPs raise G′ from 64.7 Pa to 761 Pa (≈12×); stable composite	Medium—standard PDMS soft lithography & rheometer use	3D cell culture models on microfluidic chips; tissue-like microenvironment	[15]
MatriMix composites molded into cylindrical microvessel (needle removal) and endothelialized (HUVECs)	Easy—single-vessel PDMS chip; simple and rapid insertion/removal of needle	High—ZO-1 tight junctions observed in MatriMix; improved barrier function	Good/Moderate—MatriMix slightly stiffer (200–600 Pa vs. 100–500 Pa) and lower permeability (16 vs. 40 ×10^−14^ m^2^) than collagen type I	Medium—commercial composite hydrogel + PDMS chip workflow	Vasculature-on-chip	[78]

Notes on interpretation: assessments of ‘ease of fabrication’, ‘biological fidelity’, ‘mechanical robustness’, and ‘cost’ are based solely on explicit statements in the cited papers (see last column) and mapped to qualitative ratings. No extrapolation beyond the references was performed.

## Data Availability

Not applicable.

## Abbreviations

The following abbreviations are used in this manuscript:

ADMSCs	adipose-derived mesenchymal stem cells
ALI	air–liquid interface
AM	additive manufacturing
APTES	3-aminopropyltriethoxysilane
BBB	blood–brain barrier
BET	Brunauer, Emmett, and Teller analysis
BG NPs	bioglass nanoparticles
BSA	bovine serum albumin
C6	glial cell line
CAD	computer-aided design
CHIPSs	collagen-based high-resolution internally perfusable scaffolds
CMFs	collagen type I microfibers
CNC	computer numerical control
COC	cyclic olefin copolymer
COP	cyclic olefin polymer
2D	two dimensional
3D	three dimensional
DGEA	collagen-derived peptide motif (sequence Asp-Gly-Glu-Ala)
dHAM	decellularized human amniotic
DILI	drug-induced liver injury
DMEM	Dulbecco’s Modified Eagle Medium
E_c_	compression modulus
ECM	extracellular matrix
EDC/NHS	1‑ethyl‑3‑(3‑dimethylaminopropyl)carbodiimide with N‑hydroxysuccinimide
EDS	Energy-dispersive spectroscopy
FEA	finite-element analysis
FITC	fluorescein isothiocyanate
FRESH	freeform reversible embedding of suspended hydrogels
G′	storage modulus
G″	loss modulus
GFOGER	high-affinity collagen integrin–binding motif (sequence Gly-Phe-Hyp-Gly-Glu-Arg)
GNRs	gold nanorods
GTA	glutaraldehyde
HGF	hepatocyte growth factor
HUVECs	human umbilical vein endothelial cells
KoC	kidney-on-chip
LSECs	liver sinusoidal endothelial cells
MD	molecular dynamics
MDCK	Madin–Darby canine kidney
MMP	matrix metalloproteinase
MPSs	microphysiological systems
MSCs	mesenchymal stem cells
MTT	3-(4,5-dimethylthiazol-2-yl)-2,5-diphenyltetrazolium bromide assay
MWCNTs	multi-walled carbon nanotubes
OoC	organ-on-a-chip
PAA-PG	poly(acrylic acid) photografting
PBS	phosphate-buffered saline
PC	polycarbonate
PCL	poly(ε-caprolactone)
PDA	polydopamine
PDACs	pancreatic ductal adenocarcinoma cells
PDL	poly-D-lysine
PDMS	polydimethylsiloxane
PEG	polyethylene glycol
PET	polyethylene terephthalate
PLGA	poly lactic-co-glycolic acid
PLL	poly-L-lysine
PLLA	polylactic acid
MP-ECIS	microfluidic platform designed for electrical cell–substrate impedance spectroscopy
PMMA	poly(methyl methacrylate)
PS	polystyrene
PSA	pressure-sensitive adhesive
PUMA	polyurethane methacrylate
rhBMP-2	bone morphogenic protein 2
RILD	radiation-induced liver injury
RPTECs	renal proximal tubule epithelial cells
SEM	Scanning electron microscopy
SR	swelling ratio
STOMP	suspended tissue open microfluidic patterning
TEER	transendothelial/transepithelial electrical resistance
TGF-β	transforming growth factor β
TNF-α	tumor necrosis factor alpha
TPE	thermosetting polyester
VAPOR	vascular and perfusion organ-on-a-chip reactor
VEGF	vascular endothelial growth factor
VFP	viscous finger patterned
VoCs	vessel-on-chips
